# Cathepsin S activity controls chronic stress-induced muscle atrophy and dysfunction in mice

**DOI:** 10.1007/s00018-023-04888-4

**Published:** 2023-08-17

**Authors:** Ying Wan, Limei Piao, Shengnan Xu, Xiangkun Meng, Zhe Huang, Aiko Inoue, Hailong Wang, Xueling Yue, Xueying Jin, Yongshan Nan, Guo-Ping Shi, Toyoaki Murohara, Hiroyuki Umegaki, Masafumi Kuzuya, Xian Wu Cheng

**Affiliations:** 1 Department of Cardiology and Hypertension, Yanbian University Hospital, Yanji, 133000 Jilin People’s Republic of China; 2 Jilin Provincial Key Laboratory of Stress and Cardiovascular Disease, Yanbian University Hospital, Yanji, 133000 Jilin People’s Republic of China; 3 Department of Vascular Surgery, The Second Affiliated Hospital, Zhejiang University School of Medicine, Hangzhou, 310000 Zhejiang People’s Republic of China; 4 Department of Neurology, University of Occupational and Environmental Health, Kitakyushu, Fukuoka 807-8555 Japan; 5 Institute of Innovation for Future Society, Nagoya University Graduate School of Medicine, Nagoya, Aichiken 4660855 Japan; 6 Department of Anesthesiology, Yanbian University Hospital, 1327 Juzijie, Yanji, 133000 Jilin People’s Republic of China; 7 Department of Medicine, Brigham and Women’s Hospital, Harvard Medical School, Boston, MA 02115 USA; 8 Department of Cardiology, Nagoya University Graduate School of Medicine, Nagoya, Aichi-ken 466-8550 Japan; 9 Department of Community Healthcare and Geriatrics, Nagoya University Graduate School of Medicine, Nagoya, Aichi-ken 466-8550 Japan; 10 Meitetsu Hospital, Nagoya, Aichi 451-8511 Japan

**Keywords:** Chronic stress, Skeletal muscle injury, Cathepsin S, Catabolism, Apoptosis

## Abstract

Exposure to chronic psychological stress (CPS) is an intractable risk factor for inflammatory and metabolic diseases. Lysosomal cysteinyl cathepsins play an important role in human pathobiology. Given that cathepsin S (CTSS) is upregulated in the stressed vascular and adipose tissues, we investigated whether CTSS participates in chronic stress-induced skeletal muscle mass loss and dysfunction, with a special focus on muscle protein metabolic imbalance and apoptosis. Eight-week-old male wildtype (CTSS^+/+^) and CTSS-knockout (CTSS^−/−^) mice were randomly assigned to non-stress and variable-stress groups. CTSS^+/+^ stressed mice showed significant losses of muscle mass, dysfunction, and fiber area, plus significant mitochondrial damage. In this setting, stressed muscle in CTSS^+/+^ mice presented harmful alterations in the levels of insulin receptor substrate 2 protein content (IRS-2), phospho-phosphatidylinositol 3-kinase, phospho-protein kinase B, and phospho-mammalian target of rapamycin, forkhead box-1, muscle RING-finger protein-1 protein, mitochondrial biogenesis-related peroxisome proliferator-activated receptor-γ coactivator-α, and apoptosis-related B-cell lymphoma 2 and cleaved caspase-3; these alterations were prevented by CTSS deletion. Pharmacological CTSS inhibition mimics its genetic deficiency-mediated muscle benefits. In C_2_C_12_ cells, CTSS silencing prevented stressed serum- and oxidative stress-induced IRS-2 protein reduction, loss of the myotube myosin heavy chain content, and apoptosis accompanied by a rectification of investigated molecular harmful changes; these changes were accelerated by CTSS overexpression. These findings demonstrated that CTSS plays a role in IRS-2-related protein anabolism and catabolism and cell apoptosis in stress-induced muscle wasting, suggesting a novel therapeutic strategy for the control of chronic stress-related muscle disease in mice under our experimental conditions by regulating CTSS activity.

**Supplementary Information:**

The online version contains supplementary material available at 10.1007/s00018-023-04888-4.

## Introduction

Evidence obtained in the past ~ 20 years had demonstrated that the chronic psychological stress (CPS) in modern lifestyles is closely associated with the incidence of various diseases including diabetes mellitus, cardiovascular diseases, and even hair loss [1–3]. Although recent basic and clinical studies revealed that CPS detrimentally affects human and animal skeletal muscle homeostasis [4, 5], the mechanisms that underlie these effects are largely unknown.

Muscle atrophy is often accompanied by elevations in inflammatory cytokines such as interleukin-6 (IL-6) and tumor necrosis factor-alpha (TNF-α), which induce alterations in the body’s consumption of proteins, lipids, and carbohydrates [6, 7]. Among these alterations, skeletal muscle fiber protein turnover is particular essential due to its role in maintaining the body’s muscle mass and function [8]. CPS was reported to promote cardiac and skeletal muscle inflammation and to result in fibrosis and a reduction in vascular regeneration capacity in response to ischemia [5, 9]. Other investigations showed that chronic stress induced two different nonoverlapping pathways [10]: (*i*) a ubiquitin proteasome system and (*ii*) autophagy [11] co-regulating both protein degradation and cell death by enhancing the susceptibility to apoptosis, leading to skeletal muscle mass loss.

Skeletal muscle atrophy is known to be closely associated with the inactivation of anabolic-related mammalian target of rapamycin (mTOR) signal transduction and with activation of the catabolic-related muscle RING-finger protein-1 (MuRF-1) and Muscle Atrophy F-box gene (MAFbx1) axis, both of which are major protein metabolism targets located downstream of the insulin-like growth factor 1 (IGF-1)/insulin receptor substrates (IRSs) signaling pathway [12, 13]. Insulin increased the level of IRS-2 associated with phosphatidylinositol-3 kinase (PI3K) activity, which led to increased Akt phosphorylation in skeletal muscle [14]. Insulin-induced IRS-2 down-regulation occurred via a PI3K/mTOR signaling pathway in L6 muscle cells [15]. Moreover, IRS-2 is the major adapter molecule linking the insulin receptor to the activation of protein kinase B/mitogen-activated protein kinase (MAPK) [15]. The limited number of relevant laboratory studies have shown that skeletal muscles’ IGF-1/IRSs signaling pathway was negatively regulated by stress in rats and teleost fishes [5, 10]. The reactivation of the IGF-1/IRS-2 signaling pathway might also have potential as a therapeutic strategy to prevent muscle atrophy by mitigating the protein metabolism imbalance.

The expression of members of the cathepsin family is upregulated during various forms of skeletal muscle atrophy [16]. In addition to its function as an endopeptidase, cathepsin S (CTSS) directly degrades proteins via the actions of a covalently cross-linked substrate [17]. One of our research group’s 2016 studies demonstrated that CTSS deletion confers resistance to mechanical injury via the reduction of vascular smooth muscle proliferation that was mediated by toll-like receptor (TLR)-2-dependent histone deacetylase phosphorylation [18]. We also later observed that CTSS deficiency in mice prevented chronic stress-related neointimal hyperplasia in response to injuries via the reduction of inflammation and oxidative stress production [19]. A single study documented that CTSS deficiency in *mdx*-background mice significantly increased myofiber sarcolemmal membrane stability, with greater expressions and membrane localizations of utrophin, integrins, and β-dystroglycan, which anchor the membrane to the basal lamina and underlying cytoskeletal proteins [20]. Although cathepsin K (CTSK) has been shown to be involved in cell apoptosis in vivo and in vitro [21], the roles of CTSS have remained unclear.

In the present study, we explore the role(s) of CTSS in the pathogenesis of stress-related muscle mass loss and atrophy in wildtype (CTSS^+/+^) and CTSS-knockout (CTSS^−/−^) mice with and without chronic variable stress. In a separate CTSS inhibition experiment, CTSS^+/+^ mice under stress conditions were assigned to a vehicle or a specific CTSS inhibitor (CTSS-I) treatment for 2 weeks. To investigate the underlying molecular mechanisms, after the silencing or overexpression of CTSS, C_2_C_12_ mouse myoblasts were treated with stressed serum or superoxide and subjected to biological and apoptosis assays. Based on this study’s results, we propose that CTSS is an important molecular determinant of muscle atrophy and a potential therapeutic target in individuals who are experiencing CPS.

## Materials and methods

### Animal care and use

Seven-week-old male CTSS^−/−^ (knockout [KO]) and CTSS^+/+^ (wild-type [WT]) C57BL/6 J mouse littermates weighing 22–24 g were used [19]. The mice were housed in a temperature-controlled room (22° ± 2 °C, 50% ± 5% humidity) with a 12-h light–dark cycle, with ad libitum access to food and water. The animal protocols (Protocol No. 27304) were approved by the Institution Animal Care and Use Committee of Nagoya University and performed according to the Guide for the Care and Use of Laboratory Animals published by the U.S. National Institutes of Health.

### Stress procedure

For the immobilized stress, the mouse was kept in an animal holder/stress cage (cat. no. 155-BSRR, Natsume, Seiakusho, Tokyo) for 4 h per day (from morning 9 a.m. to afternoon 1 p.m.) 7 days/week without food and water. The non-stress control mice were allowed contact with each other and left undisturbed. To prevent the mice from becoming accustomed to the restraint stress, we administered three different combinations of stressors over each week from Monday to Sunday as follows, with the order of the stress changed randomly [2]. (1) Horizontal cage and damp: We removed the sawdust from the floor of the stress cage and placed some water in the cage; the stress cage was then suspended horizontally, with the mouse’s tail in the water for 4 h once every 2 days; (2) Cage tilt: We put the mouse in the stress cage and suspended the cage at a 45° angle for 4 h once every 2 days; (3) Overnight illumination: The mouse was placed separately in a cage in a room with all-night lighting (from 21:00 to 9:00), 3 × /week.

First, for the evaluation of the effects of chronic stress on skeletal muscle, after 1 week of rest, 8-week-old CTSS^+/+^ mice were randomly assigned to a non-stress group (the non-stress control group), a 7-day-stress group (variable stress for 7 days) and a 14-day-stress group (variable stress for 14 days). Second, for the evaluation of the role of CTSS in muscle atrophy, 8-week-old male CTSS^+/+^ and CTSS^−/−^ mice were randomly assigned to a non-stress group or stress group for 2 weeks. For the CTSS inhibition experiment, CTSS^+/+^ mice receiving stress were given an intragastric administration of either vehicle (0.5% carboxymethylcellulose) or the CTSS inhibitor CTSS-I (Calbiochem, San Diego, CA) 5 mg/kg per day for 2 weeks. At the end of the stress periods, the mice were subjected to the muscle functional assay and eventually sacrificed for the biochemical and morphological analyses.

### Sample collections

At the indicated time points, all mice were anesthetized with an intraperitoneal injection of pentobarbital sodium (50 mg/kg), and both arterial blood samples from the left ventricle and muscle tissue were collected. For the biological analysis, the skeletal muscle was isolated and maintained in RNA later solution (for the gene assay) or stored at − 80 °C (for the protein assay). For the morphological analysis, after being immersed in fixative at 4 °C, the skeletal muscles were embedded in optimal cutting temperature compound (Sakura Fine-technical, Tokyo) and stored at − 20 °C. The blood was poured into an EDTA-2Na blood collection tube and centrifuged, and the plasma was collected and stored at − 80 °C. For in vivo experiments, we also isolated the serum of non-stressed and stressed mice.

### Evaluation of body weight and grip strength

The body weights of all mice were measured on Days 0, 7, and 14 after the stress period. We used a small-animal grip strength meter (Columbus Co., Largo, FL) to measure grip strength. When the forelimbs of a mouse whose tail was pulled horizontally by an examiner’s hand were no longer able to grasp the strength meter, the indicated force was deemed the maximum grip strength [22]. The grip strength was measured at least three times for each mouse on Days 0, 7, and 14, and the values were averaged as the grip strength value for each of these days. Following the functional analysis, all of the mice were euthanized by an overdose of sodium pentobarbital, and the isolated gastrocnemius muscles were subjected to biochemical and morphological analyses.

### Quantitative real-time gene expression essay

Total RNA from cells and tissues were isolated with the RNeasy Mini kit (Qiagen, Hilden, Germany) according to the manufacturer’s instructions. The mRNA was reverse-transcribed to cDNA with the SuperScript III First-Strand Synthesis System (Invitrogen, Carlsbad, CA) for a quantitative polymerase chain reaction (qPCR) assay. Quantitative gene expression was studied using the ABI7300 real-time qPCR system with Power SYBR Green PCR Master Mix (Applied Biosystems, Foster City, CA). A conventional polymerase chain reaction (PCR) protocol was also performed for targeted gene expressions, with the following conditions: 95 °C for 10 min followed by 45 cycles at 95 °C for 30 s and 55 °C for 1 min, followed 95 °C for 15 s and 60 °C for 30 s, and finally at 95 °C for 15 s. All experiments were performed in triplicate. The sequences of the primers used for all investigated genes are shown in Supplementary Table S1. The transcription of targeted genes was normalized to glyceraldehyde 3-phosphate dehydrogenase (GAPDH).

### Western blot analysis

Protein was extracted with the use of lysis buffer containing 20 mM Tris–Cl (pH 8.0), 1% Triton X -100, 150 mM NaCl, 1 mM EDTA, 0.05% SDS, 0.5% sodium deoxycholate, plus Phosphatase Inhibitor Cocktail (Roche 44,084,200) and cOmplete Protease Inhibitor Cocktail Tablets (Roche 30,819,700) from the gastrocnemius muscle and C_2_C_12_ cells. The proteins were transferred to polyvinylidene difluoride membranes and immunoreacted. They were then incubated overnight with primary antibodies against insulin receptor substrate 2 (IRS-2, #4502), protein kinase B (Akt, #2920), phospho-Akt^s473^ (p-Akt^s473^, #4060), glyceraldehyde-3-phosphate dehydrogenase (GAPDH, #5174), cleaved-caspase-3 (C-caspase-3, #9664), B-cell lymphoma-2 (Bcl-2, #3498), Desmin (D93F5), LC3B(E7X4S), (Cell Signaling Technology, Beverly, MA; 1:1,000); insulin growth factor-1 (IGF-1, #DF6096), phospho-mammalian target of rapamycin (p-mTOR, #AF3308), mTOR (#AF6308), phospho-phosphatidylinositol-3-kinase (p-PI3K, #AF3242), PI3K (#AF6242), phospho-FoxO1ɑ^ser329^ (p-FoxO1ɑ^ser329^, #AF3416), peroxisome proliferator-activated receptor-gamma (PPAR-γ) coactivator-1α (PGC-1α, #AF5395) (Affinity Biosciences, Cincinnati, OH; 1:1000); muscle atrophy Fbox (MAFbx1, #ab168372) and PPAR-γ (#ab45035) (abcam, Cambridge, MA; 1:1,000); and CTSS (sc-271619), muscle ring finger 1 (MuRF-1, sc-398608) (Santa Cruz Biotechnology, Santa Cruz, CA; 1:1,000).

The membranes were then targeted with the horseradish peroxidase (HRP)-conjugated secondary antibody at 1:10,000–1:15,000 dilution. The Amersham ECL Prime Western Blotting Detection Kit (GE Healthcare, Freiburg, Germany) was used for the determination of targeted proteins. Protein levels quantitated from western blots were normalized by loading internal controls.

### Morphometry and immunohistochemistry analyses

On stressed day 14, we prepared serial skeletal muscle cross-cryosections (4 μm thick) at a rate of 3–4 sections every 40 µm and stained them with hematoxylin and eosin (H&E). The area per muscle fiber was measured in three randomly chosen microscopic fields from six different sections in each tissue block and averaged for each mouse.

Apoptotic staining was performed as follows: the sections were subjected to terminal deoxynucleotidyl transferase-mediated dUTP nick end labeling (TUNEL) using a Fluorescein In Situ Cell Death Detection Kit (cat. no. 11684795910, Sigma-Aldrich, St. Louis, MO). For the quantification of the positive cell staining, we took 6–7 images of each section using a × 20 objective, and we counted the numbers of TUNEL^+^ cells and averaged the numbers for each mouse [16].

### Immunofluorescence and collagenolytic activity assay

To examine changes in the muscle fibers’ structural properties and healing capacity, we performed double immunofluorescence labeling of the laminin and desmin as described [16] In brief, the muscle sections were treated with a rabbit polyclonal antibody to laminin-5 (BS-7713R; Bioss, Woburn, MA) and a mouse monoclonal antibody to desmin (Clone 33; Dako, Carpinteria, CA) (1:100 for each). The sections were then visualized using Zenon rabbit and mouse IgG labeling kits (1:200; Molecular Probes, Eugene, OR) according to the manufacturer’s instructions. Staining sections were visualized with a BZ-X700 microscope (Keyence, Osaka, Japan) using 20 × or 40 × objectives. We measured the average intensity of desmin protein expression for six fields of vision in one section by using the Image J software program.

As an in vitro investigation, after cell differentiation, C_2_C_12_ myotubes were fixed with 4% paraformaldehyde for 10 min at room temperature. After three washes with phosphate-buffered saline (PBS), the slides were blocked with 5% bovine serum albumin (BSA) for 30 min. Myosin heavy chain (MHC) antibody (#ab11083, abcam; 1:500) dilution was incubated overnight at 4 °C. Alexa Fluor 488 anti-mouse secondary antibody (#A-21202, Thermo Fisher Scientific, Waltham, MA: 1:1,000) was used and incubated for 2 h at room temperature. PureBlu DAPI (#1,351,303, Bio-Rad, Hercules, CA) was used to stain the cell nuclei. In addition, we have analyzed the CTSS-mediated collagenolytic activity [19].

### Electron microscopy analysis of skeletal muscle mitochondria

Muscle samples were cut into approx. 1-mm^3^ pieces and fixed first for 24 h with 2% glutaraldehyde in 0.16 M PBS pH 7.2 and then for 1 h with 1% osmium tetroxide. The fixed tissues were dehydrated in a graded series of ethanol solutions before exposure to propylene oxide and embedding in Epon. The sections were cut at a thickness of 60–70 μm, stained with uranyl acetate and lead citrate, and observed with a transmission electron microscope (JEM-1400, JEOL, Tokyo) operating at 100 kV. The quantitation of mitochondrial number and size was performed at a magnification of 15,000 × by counting the corresponding number of pixels with the use of Image J software [23]. A total of 60–80 mitochondrial cross-sectional areas from three sections were measured for each mouse.

### Cell culture

C_2_C_12_ cells were grown in Dulbecco’s Modified Eagle’s medium (DMEM) containing 10% fetal bovine serum (FBS) and 1% antibiotics at 37 °C with 5% CO_2_. To induce the cells’ stress, when the cells reached approx. 80% confluence, they were washed twice with PBS and added to DMEM without FBS and then left overnight for cell cycle synchronization by serum starvation. The cells were then cultured in serum-free medium in the presence or absence of H_2_O_2_ at 0 and 400 µM for 24 h and subjected to biological analyses. C_2_C_12_ cells were also cultured with non-stressed serum (NS-serum) or stressed (S)-serum for 24 h and then subjected to biological analyses.

In addition, at confluence, C_2_C_12_ myoblasts were induced to fuse by a change in the original medium to medium containing 2% horse serum (called "differentiation medium" herein) as described [16]. After 7 days of differentiation, the myoblasts had lengthened, fused, and become multinucleated myotubes. After being cultured in serum-free DMEM for 6 h, the differentiated C_2_C_12_ cells were cultured in the presence of 5% NS-serum or S-serum for 24 h, and the results were subjected to the myotube diameter and biological analyses.

### The silencing of CTSS and the overexpression of CTSS

CTSS silencing was performed as described [21]. Briefly, C_2_C_12_ cells were grown on 60-mm dishes until they reached 80% confluence. Short interfering RNA against CTSS (siCTSS) solution mixed with serum-free and antibiotic-free opti-MEM medium containing Lipofectamine^®^ Transfection reagent was loaded to each well to achieve a final siRNA concentration of 100 pM. The cells were incubated at 37 °C for 48 h, and the levels of targeted gene and protein were then examined by quantitative PCR.

For the CTSS overexpression experiments, CTSS plasmid was transformed in competent *E*. *coli* cells by using the heat shock method, followed by purification using a Qiagen plasmid mini-kit. CTSS plasmid (pl-CTSS) was then transformed in C_2_C_12_ cells with the help of Lipofectamine LTX & Plus reagents (Thermo Fisher) according to the manufacturer’s instructions. Following treatments, we also analyzed the diameters of myotubes (20–30 myotubes/40 × magnification) for each section.

### Detection of ROS in vitro

The C_2_C_12_ cells transfected by siCTSS or pl-CTSS were cultured with NS-serum or S-serum for 24 h, and then the culture medium was removed, and the cells washed twice in PBS. Following treatments, each well was treated with 1 mL of DMEM without FBS consisting of 10 mol/L DCH-DA (Beyotime Company, Shanghai, China) and further cultured at 37℃ for 15 min. At the same time, the probe dish (Agilent Technologies 103,059–000) was hydrated overnight without CO_2_. The cells were incubated in an OCR buffer for 45 min and tested using the Agilent Technologies 103,010–100 kit.

### Evaluation of mitochondrial respiration

An Agilent Seahorse XFp Analyzers was warmed more than 5 h, the C_2_C_12_ cells transfected by siCTSS or pl-CTSS were cultured with NS-serum or S-serum for 24 h, then cells were treated to different purposes before being plated in Seahorse assay cell culture dishes overnight (Agilent Technologies 103,025–100). At the same time, the probe dish was hydrated overnight in the absence of CO_2_ (Agilent Technologies 103,059–000). Then the C_2_C_12_ cells were incubated in an OCR buffer for 45 min and tested using the Agilent Technologies (103,010–100) kit following the instructions.

### Cell apoptosis assay

The C_2_C_12_ cells transfected by siCTSS or pl-CTSS were cultured in serum-free DMEM containing 400 µM H_2_O_2_ for 24 h and then subjected to TUNEL staining following the manufacturer’s instructions (Sigma-Aldrich). Transfected cells were also cultured with NS-serum or S-serum for 24 h and then subjected to a TUNEL staining assay [16]. The apoptotic cells in the muscles were also evaluated by TUNEL staining.

### Statistical analyses

The data are expressed as the mean ± SEM (standard error of the mean). Student’s t tests (for comparisons between two groups) and a one-way analysis of variance (ANOVA) for comparisons of three or more groups followed by Tukey’s post hoc tests were used for the statistical analyses. The nonparametric Kruskal–Wallis test (Tukey-type multiple comparison) was used for the ANOVA of the gene expression data. Body weight and grip strength data were subjected to a two-way repeated-measures ANOVA and Bonferroni post hoc tests. Origin software ver.6 1 was used, and p values < 0.05 were considered significant. All parameter calculations were conducted by two observers blinded to the treatment of the mice.

## Results

### Chronic stress enhanced the muscle injury and dysfunction associated with skeletal muscle morphological and biological alterations

**Fig. 1:**
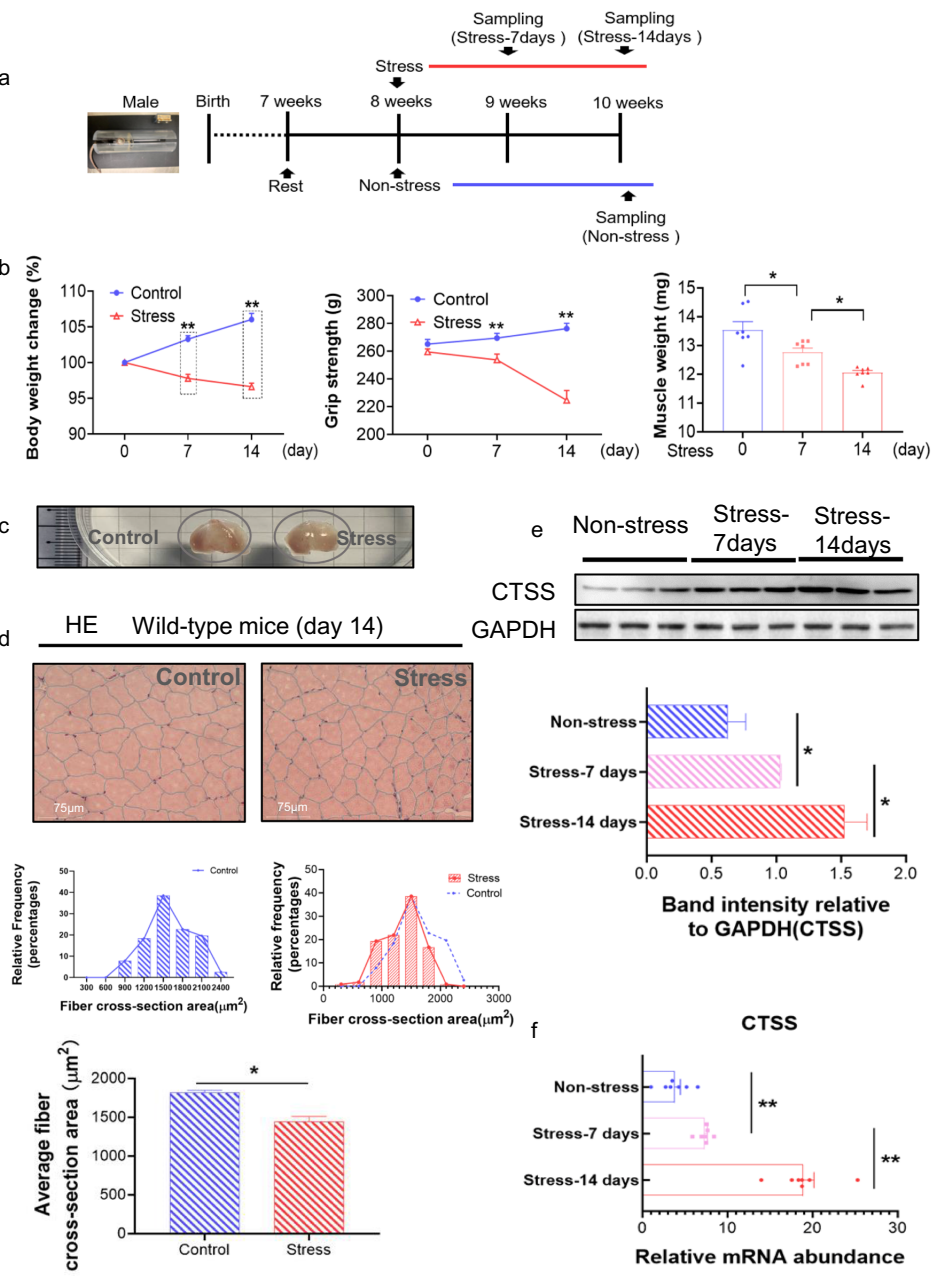
Chronic stress accelerated skeletal muscle mass loss and dysfunction. **a**: The mouse immobilized stress model. At the indicated time points after being subjected to variable stress, mice were sacrificed for biochemical and morphological analyses. **b**: Left panel: The body weight changes of CTSS^+/+^ mice with non-stress (Control) and a 14-day stress period (Stress) (*n* = 7 each). Middle panel: Grip strength analysis of Control and Stress mice (*n* = 7). Right panel: Gastrocnemius (GAS) muscle weights of Control and Stress mice (*n* = 7). **c**: Representative images of GAS muscle shape after sampling. **d**: Representative images of hematoxylin and eosin (H&E) staining of the GAS muscle sections of wildtype (CTSS^+/+^) mice. Scale bar: 75 µm and quantitative data showing the cross-sectional area of a GAS fiber (*n* = 5). **e**: Representative immunoblotting images and quantitative data for CTSS in GAS muscles at Days 7 and 14 after stress (*n* = 3). **f**: RT-qPCR analysis of CTSS in GAS muscle (*n* = 7, each group). The data are mean ± SEM, and p-values were determined by a two-way repeated measures ANOVA and Bonferroni’s post hoc tests (b: left panel and middle panel, e,f). One-way ANOVA followed by Tukey’s post hoc tests (b: right panel) or unpaired Student’s t-test (d). Control: CTSS^+/+^ control mice, Stress: CTSS^+/+^ 14-day-stressed mice. *p < 0.05; **p < 0.01; N.S, not significant

The variable stress protocol has been often used to evaluate CPS-related harmful effect on cardiovascular and other organs [2, 24]. For the investigation of the impacts of chronic stress on the expression of CTSS and muscle disorder, 8-week-old mice were subjected to a 2-week variable stress protocol (Fig. 1a). As shown in Fig. 1b, the stress significantly reduced the body weight of the mice in a time-dependent manner. As anticipated, stressed mice showed a marked reduction in grip strength (less > 10%) and gastrocnemius (GAS) muscle weights (loss > 11%) (Fig. 1b, c). The quantitative data of H&E staining also showed a marked reduction in the myofiber cross-sectional area in the GAS muscles of 14-day stressed mice (average area reduction > 20%) compared to the non-stressed mice (Fig. 1d). The qPCR and western blotting data showed that (*i*) the expression of CTSS was the most sensitive to chronic stress induction compared to other members of the cathepsin family, and (*ii*) the CTSS protein expression in the stressed muscles of CTSS^+/+^ mice presented in a time-dependent manner (Fig. 1e, f, S1e).

We also observed a marked reduction of positive desmin staining signal in the stressed muscles of CTSS^+/+^ mice (reduction > 20%, Suppl. Fig. S1a,c). The qPCR and TUNEL staining revealed that the stress resulted in (*i*) an increase in the levels of oxidative stress (gp91^phox^ and p47^phox^)-related and inflammation (TNF-α, ICAM-1, MCP-1, TLR-4 and MyD88)-related mRNA in a time-dependent manner, (*ii*) an increase in the numbers of TUNEL^+^ cells, and (*iii*) a time-dependent decrease in the levels of mitochondrial biogenesis-related (PPAR-γ and PGC-1α) genes (Suppl. Fig. S1b-e).

### CTSS deletion prevented the muscle wasting and dysfunction associated with skeletal muscle protein turnover

**Fig. 2:**
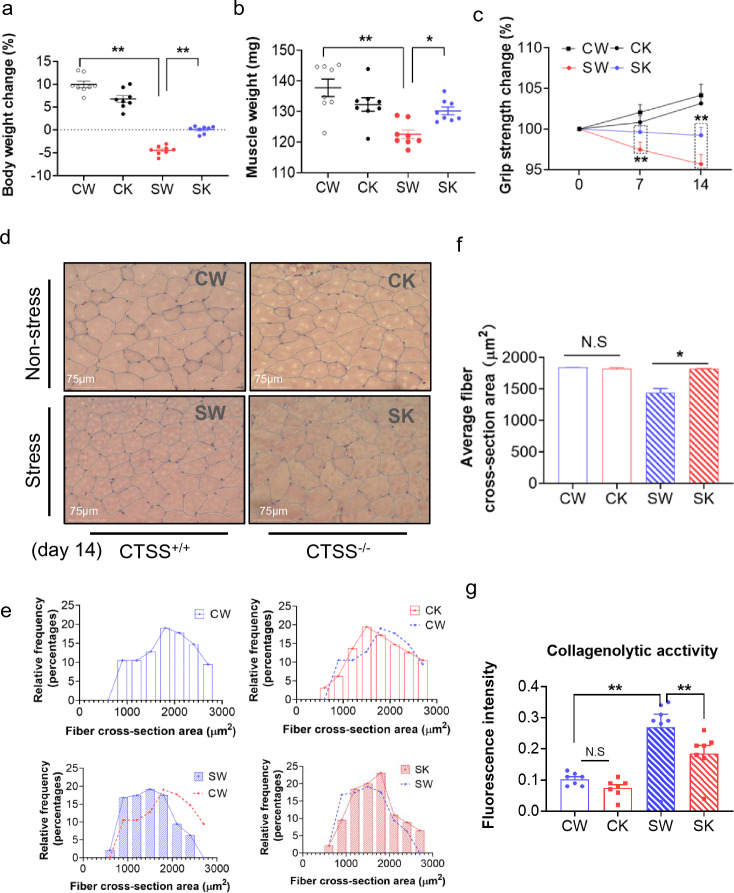
CTSS deficiency protected against stress-related muscle damage. **a**: The body weights and (**b**) GAS muscle weights of the four groups of mice (CW: CTSS^+/+^ control mice, CK: CTSS^−/−^ control mice, SW: 14-day-stressed CTSS^+/+^ mice, SK: 14-day-stressed CTSS^−/−^ mice; *n* = 8 each) on the 14th day after stress. **c**: The measurements of all four limbs’ grip strength in the four groups of mice at the indicated points (*n* = 8, each group). **d**: Representative H&E images of GAS muscle sections. **e–f**: Quantitative data showing the cross-sectional area of a GAS fiber (*n* = 5). **g**: The FITC-labeled type I collagen wee applied to evaluate the levels of CTSS-mediated collagenolytic activity using a specific CTSS inhibitor (CTSS-I) in the skeletal muscles of the non-stressed and stressed mice. Scale bar: 75 µm. The data are mean ± SEM, and p-values were determined by two-way repeated measures ANOVA and Bonferroni’s post hoc tests (c) or a one-way ANOVA followed by Tukey’s post hoc tests (a,b,f). *p < 0.05; **p < 0.01; N.S, not significant

**Fig. 3:**
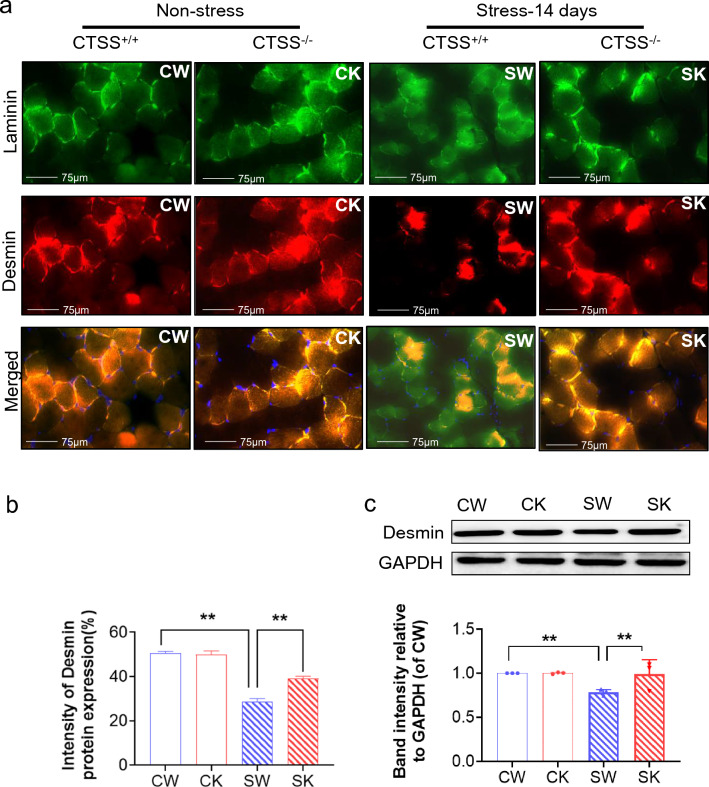
CTSS deletion alleviated muscle microstructure repair in mice subjected to stress. **a**, **b**: Representative immunofluorescence images and quantitative data for the intensity of desmin protein expression in the four experimental groups at day 14 after stress (*n* = 5). Scale bar: 75 µm. **c**: Representative immunoblotting images and quantitative data for Desmin and GAPDH protein in the lysates from the four groups (*n *= 3). The data are mean ± SEM, and *p*-values were determined by a one-way ANOVA followed by Tukey’s post hoc tests (**b**). CW: CTSS^+/+^ control mice, CK: CTSS^−/−^ control mice, SW: 14-day-stressed CTSS^+/+^ mice, SK: 14-day-stressed CTSS^−/−^ mice. *p < 0.05; **p < 0.01; N.S, not significant

As shown in Fig. 2a, CTSS deficiency resulted in a significantly lower rate of body weight change (over > 6%) compared to that in the 14-day-stressed CTSS^+/+^ mice. The GAS weights of the latter group of mice were (loss > 10%) lower than the corresponding weights in the non-stressed CTSS^+/+^ mice (Fig. 2b). In contrast, the 14-day-stressed CTSS^−/−^ mice presented almost no loss (less < 1.3%, *p* > 0.05) of muscle mass compared to the non-stressed CTSS^−/−^ mice. The grip strength monitoring indicated that the grip strength declined (less > 8%) in parallel with the muscle weights in the 14-day-stressed CTSS^+/+^ mice; these changes were prevented by CTSS deletion (Fig. 2b, c). Likewise, the quantitative data revealed by the H&E staining analysis showed that the GAS muscle-fiber cross-sectional area was higher in the 14-day-stressed CTSS^−/−^ mice (average area over > 25%) than the 14-day-stressed CTSS^+/+^ mice (Fig. 2d, f). Quantitative data revealed that the stressed CTSS^+/+^ muscles had increased the levels of the CTSS-mediated activity (over 2.6-fold) than that of the muscles of the control mice; this change was reversed by 25% in stressed CTSS^−/−^ muscles (Fig. 2g). As shown in Fig. 3, the 14-day-stressed CTSS^−/−^ mouse muscles had markedly higher intracellular intensity of desmin protein expression (increase > 10%) compared to the 14-day-stressed CTSS^+/+^ muscles, which suggests that CTSS deletion preserved the structural properties of the skeletal muscle and restored muscle repair in response to stress. Taken together, these observations indicate that CTSS deletion alleviated muscle wasting and dysfunction in mice subjected to chronic variable stress. However, there were no significant differences in the body weight change rate, GAS mass, or myofiber size between the mice of the two genotypes at the basal conditions (Figs. 2, 3).

**Fig. 4:**
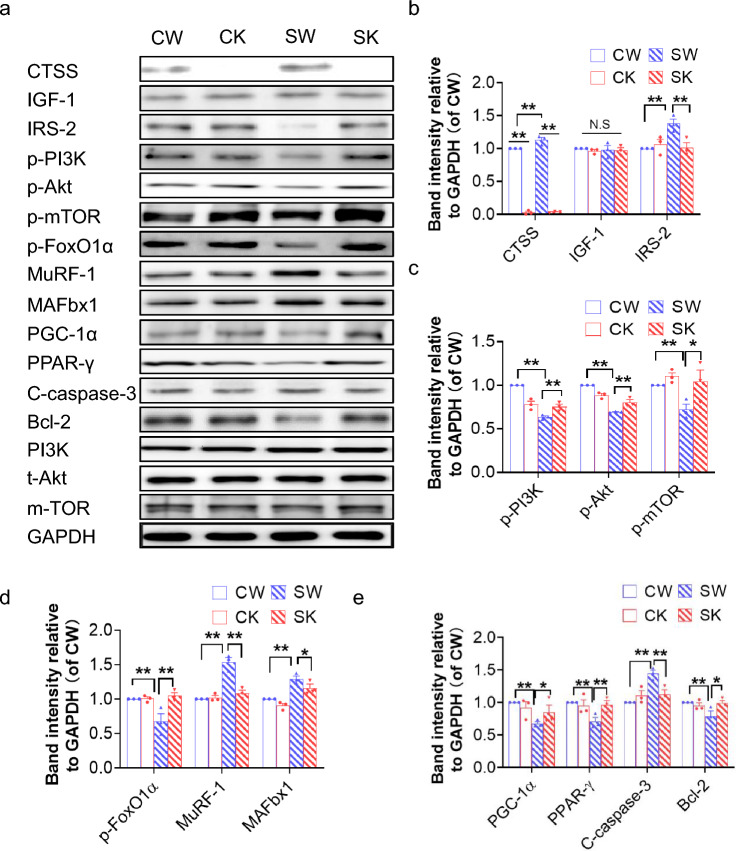
CTSS deficiency ameliorated stress-related anabolic and catabolic molecular alterations. **a–e**: Representative immunoblotting images and quantitative data for CTSS, IGF-1, IRS-2, p-PI3K, p-Akt, p-mTOR, p-FoxO1α, MuRF-1, MAFbx1, PGC-1α, PPAR-γ, C-caspase-3, and Bcl-2 in GAS muscles at Day 14 after stress (*n* = 3). Data are mean ± SEM, and *p*-values were determined by a one-way ANOVA followed by Bonferroni post hoc tests (**b**–**e**). CW: CTSS^+/+^ control mice, CK: CTSS^−/−^ control mice, SW: 14-day-stressed CTSS^+/+^ mice, SK: 14-day-stressed CTSS^−/−^ mice. *p < 0.05; **p < 0.01; N.S, not significant

The results presented in Fig. 4a, b show that the intracellular IRS-2 protein levels were reduced in the muscle isolated from 14-day-stressed CTSS^+/+^ mice. In the CTSS^−/−^ mice, as in the 14-day-stressed CTSS^−/−^ mice, even though the stress-loaded muscles still contained an abundant amount of IRS-2 proteins (Fig. 4a, b), the levels of IGF-1, which is the upstream protein of IRS-2, did not change significantly in any of the four experimental groups (Fig. 4a, b). Insulin/IGF-1 signal was impaired in the chronic stress conditions, which might cause insulin resistance, hyperglycemia, a prothrombotic state, and diabetic skeletal muscle disorder.^5^ We also observed that stress resulted in decreases in the levels of p-PI3K, p-Akt, and p-mTOR proteins and GLUT-4 gene located downstream of the IGF-1/IRS-2 signaling pathway, and that CTSS^−/−^ reversed these molecule changes after stress (Fig. 4a–c, Suppl. Fig. S2b). Akt phosphorylates FoxO transcription factors to decrease ubiquitination-related gene expression, leading to a mitigation of muscle atrophy.^25, 26^ The results of our western blotting analysis revealed that the stress also caused a change in the levels of p-FoxO1, MuRF-1 and MAFbx1 proteins, and these changes were rectified by CTSS deletion (Fig. 4a–d). These results raised the possibility that increased CTSS might cause an imbalance of skeletal muscle protein anabolism and catabolism and insulin resistance via the modulation of the IRS-2 signaling pathway in stressed mice.

**Fig. 5:**
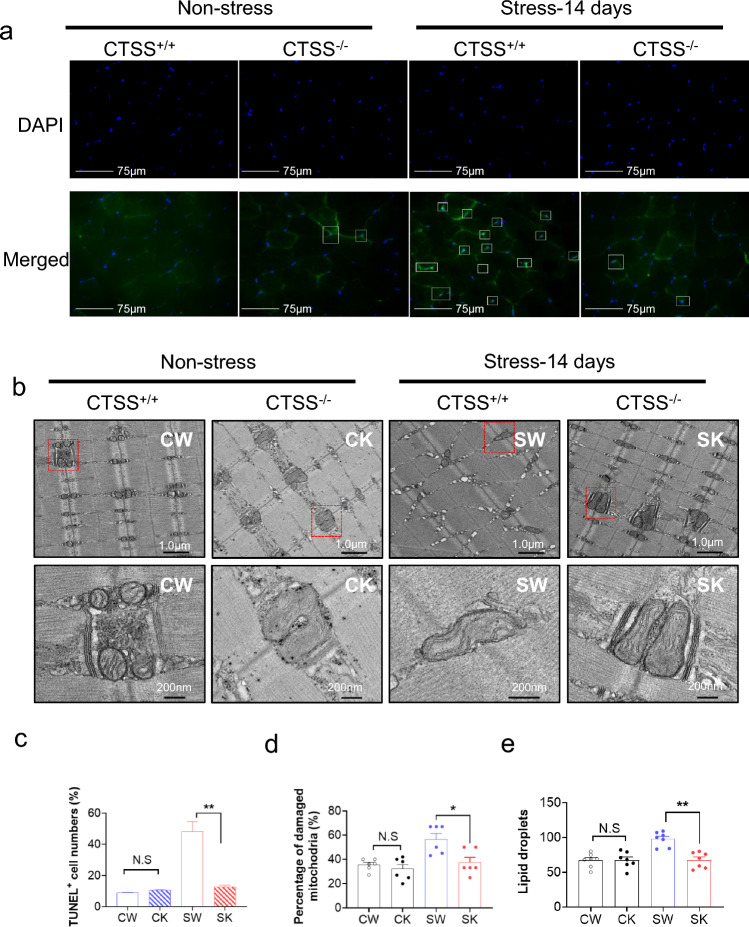
CTSS deletion alleviated muscle apoptosis and mitochondrial morphological changes in mice subjected to stress. **a**: Representative TUNEL staining used to assess the content of apoptotic cells*. Yellow arrows:* TUNEL-positive cells. **c**: Quantitative data for TUNEL-positive cells (*n* = 6). Scale bar: 75 µm. **c**–**e**: Representative images. **b:** Transmission electron microscopic images combined with quantitative data showing the percentage of damaged mitochondria (**d**) and the number of lipid droplets (**e**). Scale bars: 1.0 µm and 200 nm (*n* = 5). Data are mean ± SEM, and p-values were determined by one-way ANOVA followed by Tukey’s post hoc tests (b,d,e). CW, etc. are explained in the earlier figure legends. *p < 0.05; **p < 0.01; *NS*, not significant

In addition, as expected, at day 14 after stress, CTSS deficiency mitigated the harmful changes in the levels of CTSK, CTSL, cystatin C, gp91^phox^, p47^phox^, TNF-α, ICAM-1, MCP-1, TLR-4 and MyD88 in stressed muscles (Suppl. Fig. S2a,c,d). CTSS deficiency also improved the levels of C-caspase-3 and Bcl-2 (Fig. 4a–e). The GAS muscle from the 14-day-stressed CTSS^+/+^ mice exhibited a significantly higher proportion of apoptotic cells compared to that of the CTSS^+/+^ control mice; this effect was blocked by CTSS deletion (Fig. 5a, c). These observations thus indicate that CTSS might act as a key mediator of the harmful oxidative stress, inflammation, and apoptosis that occur in mice in response to chronic stress injury.

### CTSS^−/−^ improved muscle mitochondrial damage and biogenesis

We used transmission electron microscopy to evaluate the quality of mitochondria in GAS muscle (Fig. 5b). Compared to the non-stressed CTSS^+/+^ mice, accumulations of enlarged and abnormally shaped or swollen mitochondria were observed in the muscles of the 14-day-stressed CTSS^+/+^ group. The numbers of lipid droplets and the percentage of damaged mitochondria were significantly lower in the 14-day-stressed CTSS^−/−^ mice compared to the 14-day-stressed CTSS^+/+^ mice (Fig. 5d, e). Consistently, the qPCR and western blotting analysis showed that compared to the 14-day-stressed CTSS^+/+^ mice, the 14-day-stressed CTSS^−/−^ mice had significantly increased levels of PPAR-γ and PCG-1α protein and gene (Fig. 4a, e and Suppl. Fig. S2b), indicating that there is negative feedback from damaged mitochondria to PGC1a which lowers its expression and may contribute to the skeletal muscle atrophy and dysfunction that we observed in the CTSS^+/+^ mice under stress conditions.

### Pharmacological inhibition of CTSS prevented chronic stress-related muscle injury and dysfunction

**Fig. 6:**
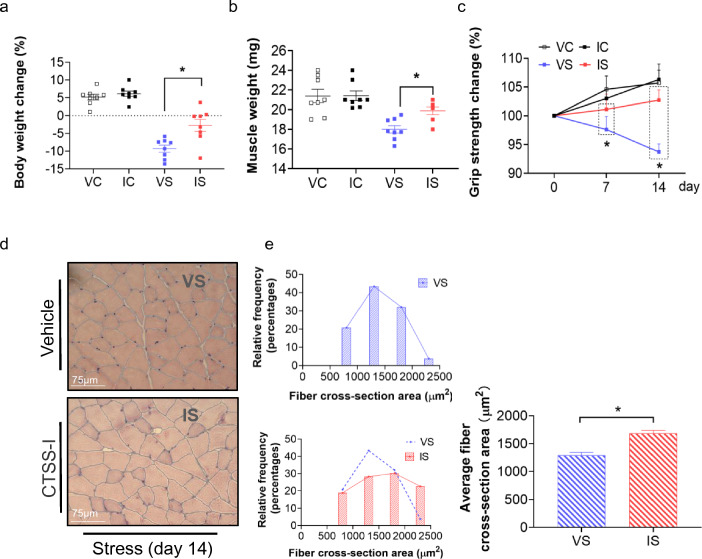
The cathepsin S inhibitor (CTSS-I) alleviated stress-related skeletal muscle injury and dysfunction. **a**, **b**: The body weights and GAS muscle weights of the four groups of mice (*n* = 8, each group). **c**: The measurements of all four limbs’ grip strength in four groups of mice at the indicated time points (*n* = 8 each). **d**, **e**: Representative H&E images and quantitative data for the cross sections of GAS muscle fibers harvested from the four groups of mice on Day 14. Scale bar: 75 µm. The data are mean ± SEM, and *p*-values were determined by a one-way ANOVA followed by Tukey’s post hoc tests (**a**, **b**), unpaired Student’s *t* test (E right panel), or two-way repeated measures ANOVA and Bonferroni’s post hoc tests (**c**). VC: CTSS^+/+^ loaded vehicle + non-stress, IC: CTSS^+/+^ loaded CTSS-I + non-stress, VS: CTSS^+/+^ loaded vehicle + stress, IS: CTSS^+/+^ loaded CTSS-I + stress. *p < 0.05; **p < 0.01; N.S, not significant

The data in Fig. 6a–c demonstrate that the body weight, grip strength, and GAS muscle weights were decreased in the CTSS^+/+^ vehicle + stress-loaded mice, and these changes were reversed in the CTSS^+/+^ CTSS-I + stress-loaded mice. CTSS inhibition also preserved the muscle fiber size (Fig. 6d, e). Similar to the deletion of CTSS, the pharmacological inhibition of CTSS prevented skeletal muscle laminin disorder and desmin protein loss as well cell apoptosis in CTSS^+/+^ mice loaded with CTSS-I + stress (Suppl. Fig. S3a-e). As anticipated, we observed that CTSS inhibition exerted beneficial effects on the levels of the investigated genes (CTSK, CTSL, cystatin C, gp91^phox^, p47^phox^, GLUT-4, PGC-1α, PPAR-γ, TNF-α, ICAM-1, MCP-1, TLR-4 and MyD88) and proteins (IRS-2, p-PI3K, p-Akt, p-mTOR, p-FoxO1α, C-caspase-3, Bcl-2, PGC-1α, PPAR-γ, MuRF-1, and MAFbx1) (Suppl. Figs. S3f, S4). CTSS inhibition thus appears to exert a musculoprotective effect against stress.

### Genetic modifications of CTSS modified the oxidative stress and S-serum-induced C_2_C_12_ protein metabolism and apoptosis

The TUNEL staining showed marked TUNEL^+^ apoptotic cells in response to the treatment with 400 µM H_2_O_2_ (Suppl. Fig. S5a,b). Next, C_2_C_12_ cells treated with 0, 200, or 400 µM H_2_O_2_ were subjected to western blotting and qPCR assays. We observed harmful changes in the levels of CTSS, IRS-2, p-Akt, p-FoxO1α, MuRF-1, C-caspase-3, and Bcl-2 in C_2_C_12_ cells treated with H_2_O_2_ at the indicated concentrations (Suppl. Fig. S5c-d). As shown in Supplementary Fig S5e, compared to the other members of the cathepsin family, the mRNA expression for CTSS was the most sensitive to H_2_O_2_ induction, in a dose-dependent manner. We observed that siCTSS suppressed the CTSS gene and protein expressions in C_2_C_12_ cells treated with and without 400 µM H_2_O_2_ (Suppl. Fig. S6a-b). The TUNEL staining and western blotting assays indicated that cell apoptosis induced by H_2_O_2_ occurred in parallel with the negative changes in the levels of CTSS, IRS-2, p-Akt, p-FoxO1α, MuRF-1, C-caspase-3, and Bcl-2; these effects were reversed by CTSS silencing (Suppl. Fig. S6e-f). In contrast, pl-CTSS caused enhanced CTSS gene and protein expressions in C_2_C_12_ cells treated with and without 400 µM H_2_O_2_ (Suppl. Fig. S7a-b). Representative immunofluorescent images indicated successful GPF-labeled CTSS plasmid transfection into C_2_C_12_ cells (Suppl. Fig. S7c).

The representative MHC staining showed that (*i*) CTSS silencing prevented S-serum- and H_2_O_2_-induced myotube atrophy, and (*ii*) CTSS overexpression induced myotube atrophy (Suppl. Figs. S6c, S7d). As shown in Supplementary Figure S7e, f, pl-CTSS accelerated cell apoptosis, accompanied by the negative changes in the levels of CTSS, IRS-2, p-Akt, p-FoxO1α, MuRF-1, C-caspase-3, and Bcl-2 (Suppl. Figs. S7-8), indicating that CTSS might modulate H_2_O_2_-induced apoptosis and protein metabolism via IRS-2-mediated apoptosis- and proteosome-related molecule changes in C_2_C_12_ cells [25–28].

**Fig. 7:**
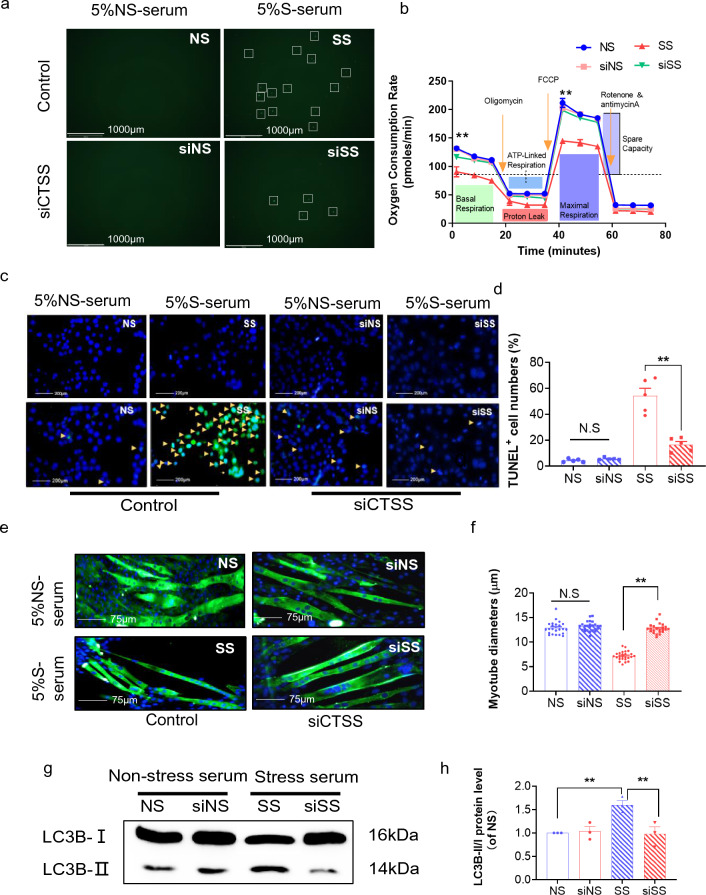
CTSS deletion mitigated S-serum-induced C_2_C_12_ myoblast ROS produced, reduction in mitochondrial respiration, cell apoptosis and myotube atrophy. C_2_C_12_ cells were treated with siCTSS and non-targeting control RNA for 48 h and then cultured in 5% NS-serum or 5% S-serum for 24 h. **a**: Representative fluorescent microscopy images showing intracellular ROS in four experimental groups. Scale bar: 1000 µm. **b**: The oxygen consumption rate measured using the Seahorse XFp assay in four groups of cells. **c**: Representative image of TUNEL immunofluorescence (**c**) and combined quantitative data (**d**) show the numbers of TUNEL^+^ apoptotic cells in the four experimental groups (*n* = 5). *Yellow arrows:* TUNEL^+^ cells. Scale bar: 200 µm. **e**: Myotubes treated with siCTSS or non-targeting control were cultured in 5% S-serum or NS-serum for 72 h and then subjected to immunofluorescence staining for MHC (NS, SS, siNS, siSS). **f**: Quantification of the mean diameter of four groups. **g**: Representative immunoblotting images and (**h**) quantitative data for LC3B-II/I in the lysates from the four groups (*n* = 3). Data are mean ± SEM, and *p*-values were determined by one-way ANOVA followed by Tukey’s post hoc tests (b,d,f,h). NS: C_2_C_12_ cells treated non-targeting control RNA were cultured in 5% NS-serum for 24 h, siNS: CTSS-silenced C_2_C_12_ cells cultured in 5% NS-serum for 24 h, SS: C_2_C_12_ cells treated non-targeting control RNA were cultured in 5% S-serum for 24 h, siSS: CTSS-silenced C_2_C_12_ cells cultured in 5% S-serum for 24 h. *p < 0.05; **p < 0.01; N.S, not significant

**Fig. 8:**
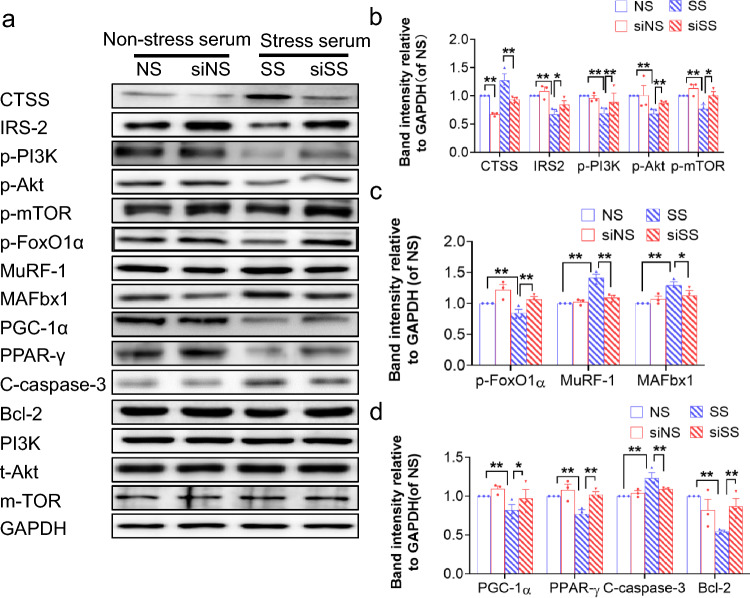
CTSS deletion ameliorated S-serum-induced C_2_C_12_ myoblast anabolic and catabolic molecular alterations. **a-d**: Representative immunoblotting images and quantitative data for CTSS, IRS-2, p-PI3K, p-Akt, p-mTOR, p-FoxO1α, MuRF-1, MAFbx1, PGC-1α, PPAR-γ, C-caspase-3, and Bcl-2 in the lysates from the four groups (*n* = 3). Data are mean ± SEM, and *p*-values were determined by one-way ANOVA followed by Tukey’s post hoc tests (**b**–**d**). NS: C_2_C_12_ cells treated non-targeting control RNA were cultured in 5% NS-serum for 24 h, siNS: CTSS-silenced C_2_C_12_ cells cultured in 5% NS-serum for 24 h, SS: C_2_C_12_ cells treated non-targeting control RNA were cultured in 5% S-serum for 24 h, siSS: CTSS-silenced C_2_C_12_ cells cultured in 5% S-serum for 24 h. *p < 0.05; **p < 0.01

**Fig. 9:**
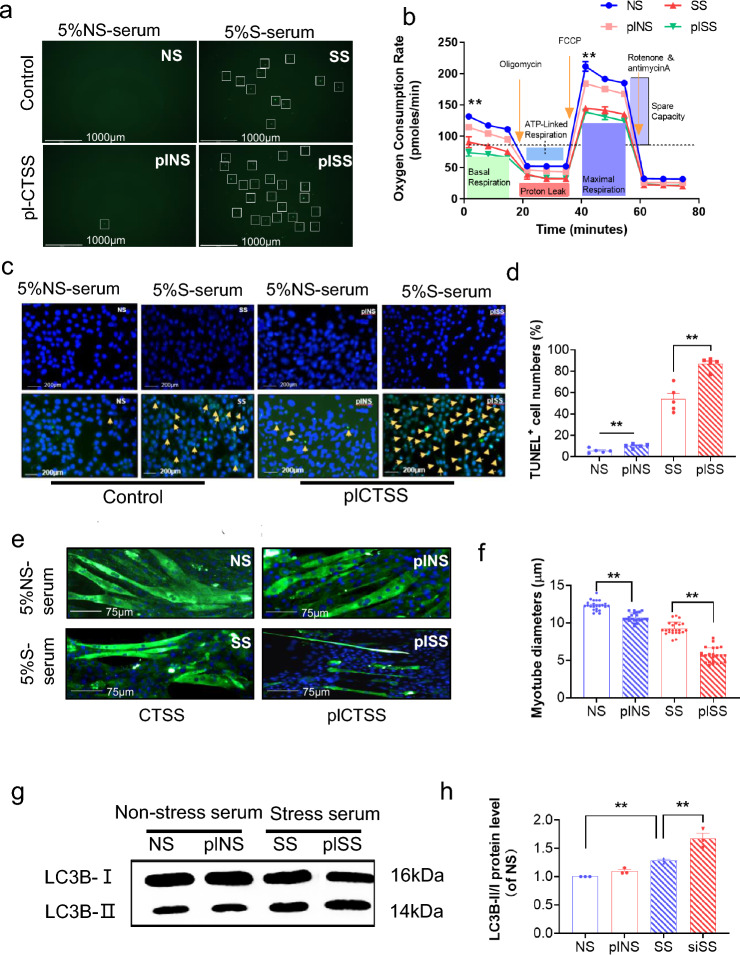
CTSS overexpression stimulated S-serum-induced C_2_C_12_ cell ROS produced, reduction in mitochondrial respiration, cell apoptosis and myotube atrophy. C_2_C_12_ cells were treated with pl-CTSS and empty plasmid for 48 h, cultured in 5% NS-serum or 5% S-serum for 24 h, and then subjected to Detection of ROS. **a**: Representative fluorescent microscopy images showing intracellular ROS in the four experimental groups. Scale bar: 1000 µm. **b**: The oxygen consumption rate measured using the Seahorse XFp assay in four groups of cells. **c**, **d**: Representative image of TUNEL immunofluorescence (**c**) and combined quantitative data (**d**) show the numbers of TUNEL^+^ apoptotic cells in the four experimental groups (*n* = 5)*. Yellow arrows:* TUNEL^+^ cells. Scale bar: 200 µm. **e**: Myotubes treated with pl-CSTT or empty control plasmid were cultured in 5% S-serum for 72 h and then subjected to immunofluorescence staining for MHC (NS, SS, plNS, plSS). **f**: Quantification of the mean diameter of four groups. **g**: Representative immunoblotting images and (**h**) quantitative data for LC3B-II/I in the lysates from the four groups (*n* = 3). Data are mean ± SEM, and *p*-values were determined by one-way ANOVA followed by Tukey’s post hoc tests (**b**, **d**, **f**, **h**). NS: C_2_C_12_ cells treated non-targeting control RNA were cultured in 5% NS-serum for 24 h, siNS: CTSS-silenced C_2_C_12_ cells cultured in 5% NS-serum for 24 h, SS: C_2_C_12_ cells treated non-targeting control RNA were cultured in 5% S-serum for 24 h, siSS: CTSS-silenced C_2_C_12_ cells cultured in 5% S-serum for 24 h. *p < 0.05; **p < 0.01; *NS*, not significant

**Fig. 10:**
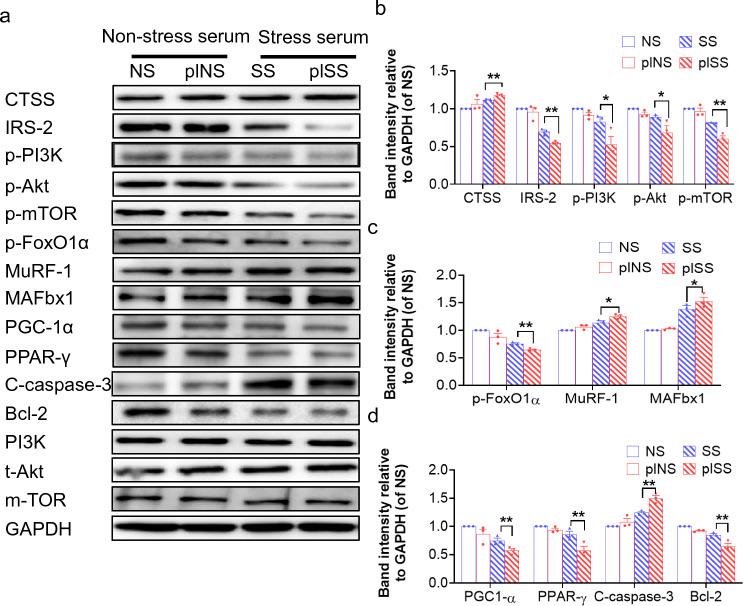
CTSS overexpression stimulated S-serum-induced C_2_C_12_ myoblast anabolic and catabolic molecular alterations. **a-d**: Representative immunoblotting images and quantitative data for CTSS, IRS-2, p-PI3K, p-Akt, p-mTOR, p-FoxO1α, MuRF-1, MAFbx1, PGC-1α, PPAR-γ, C-caspase-3, and Bcl-2 in the whole-cell lysates of the groups (*n* = 3). The data are mean ± SEM, and *p*-values were determined by one-way ANOVA followed by Tukey’s post hoc tests (**b**–**d**). NS: C_2_C_12_ cells treated with empty control plasmid were cultured in 5% NS-serum for 24 h, plNS: CTSS-overexpressed C_2_C_12_ cells cultured in 5% NS-serum for 24 h, SS: C_2_C_12_ cells treated with empty control plasmid were cultured 5% S-serum for 24 h, plSS: CTSS-overexpressed C_2_C_12_ cells cultured in 5% S-serum for 24 h. *p < 0.05; **p < 0.01

As a final step to examine the relationship between stressed serum and muscle damage with the CTSS genetic modification, we studied S-serum-induced reactive oxygen species apoptosis protection and mitochondrial respiration in genetically modified C_2_C_12_ cells. We observed that CTSS silencing lowered S-serum-induced intracellular ROS production and rescued mitochondrial respiration in C_2_C_12_ cells in response to S-serum (Fig. 7a, b). CTSS silencing resulted in a decrease in C_2_C_12_ cell apoptosis (Fig. 7c, d) and a beneficial effect on the levels of CTSS protein as well as the levels of the protein anabolism (IRS-2, p-PI3K, p-Akt, p-mTOR)- protein catabolism (p-FoxO1α, MuRF-1, MAFbx1)-, mitochondrial biogenesis (PGC-1α, PPAR-γ)-, and apoptosis (C-caspase-3 and Bcl-2)-related proteins in S-serum-treated C_2_C_12_ cells (Fig. 8). Consistently, CTSS silencing lowered stress-induced elevation of the LC3B-II to LC3B-1 ratio (Fig. 7g, h). As anticipated, CTSS overexpression produced a harmful effect on intracellular ROS production, mitochondrial respiration, cell apoptosis and accelerated these molecular harmful alterations (Figs. 9a–d, g, h, 10). The MHC staining showed that silencing CTSS prevented S-serum-induced myotube atrophy, whereas overexpressing CTSS induced myotube atrophy (Figs. 7e–f, 9e–f). Taken together, these observations provide a mechanistic explanation of the involvement of CTSS in skeletal muscle protein turnover and atrophy in vitro.

## Discussion

The above-described experiments focused on possible roles of CTSS in skeletal muscle atrophy and dysfunction in mice under variable stress conditions. The significant finding of this study is that mice lacking the CTSS gene were resistant to chronic stress-induced skeletal muscle mass loss and functional decline. At the molecular level, CTSS deficiency was observed to prevent (*i*) IRS-2 protein reduction and the downstream protein anabolism-related Akt/mTOR signaling inactivation and catabolism-related MuRF-1/MAFbx1 activation; (*ii*) TNF-α/TLR-4-MyD88-mediated inflammation and NADPH oxidase-mediated oxidative stress production; (*iii*) Bcl-2/caspase-3 imbalance-related apoptosis; and (*iv*) PPAR-γ/PGC-1α inactivation-mediated mitochondrial damage. The pharmacological inhibition of CTSS also exerted a musculoprotective effect in mice in response to stress. The in vitro results demonstrated that the silencing and overexpression of CTSS respectively increased and decreased the levels of IRS-2 and its downstream anabolic and catabolic signaling and cellular apoptotic events in C_2_C_12_ cells under stressed serum and oxidative stress conditions, providing evidence and a mechanistic explanation of the participation of CTSS in IRS-2 signaling in stress-induced skeletal muscle wasting and dysfunction (Suppl. Fig. S8).

It was reported that cathepsin is highly expressed in damaged muscles [16]. In the present study, we examined changes in CTSS during stress-induced muscle atrophy and loss of function, and we observed a time-dependent up-regulation in muscle. The levels of CTSS were higher than those of other cathepsins (CTSK, CTSL, and cystatin C) at the follow-up points. The CTSS gene and protein expressions were also sensitive to S-serum and H_2_O_2_ induction in C_2_C_12_ cells. The IGF-1/IRS-2-Akt pathway has been shown to induce hypertrophy by activating protein synthesis [15]. The same pathway can also negatively regulate muscle atrophy markers (MuRF-1 and MAFbx1) that induce the degradation of crucial muscle proteins [29]. In our model, the IRS-2 content was decreased, and imbalanced protein metabolism was manifested as the reduction of anabolism-related signal molecule phosphorylation levels (p-PI3K, p-Akt, p-mTOR and p-FoxO1α) and the enhancement of catabolism-related molecule levels (MuRF-1 and MAFbx1) on the 14th day after the stress period. In the present experiments, the genetic modification and pharmacological modification of CTSS activity both retarded skeletal muscle mass loss and dysfunction by the mitigation of those protein metabolism-related molecule harmful changes. In cellular experiments, we observed that CTSS silencing resulted in increased levels of IRS-2, p-PI3K, p-Akt, p-mTOR, and p-FoxO1α and decreased levels of MuRF-1 and MAFbx1 in C_2_C_12_ cells under S-serum or oxidative stress conditions, whereas CTSS overexpression produced opposite effects in the same conditions. Moreover, CTSS silencing prevented myotube atrophy, whereas CTSS overexpression accelerated it. Taken together, these data indicate that the CTSS inhibition-mediated skeletal muscle protective effect is likely attributable, at least in part, to a rectification of the skeletal muscle protein anabolism and catabolism imbalance produced under our experimental stress conditions.

Chronic stress has been shown to enhance the inflammatory response in different tissues (e.g., adipose and vascular tissues) [30, 31]. We observed that chronic stress resulted in an increase in the expressions of inflammatory gene (i.e., TNF-α, ICAM-1, MCP-1, TLR-4 and MyD88) and a decrease in the grip strength of all four limbs of the mice. Pro-inflammatory effects of these molecules on the processes of muscle and other tissue remodeling and dysfunction have been sufficiently demonstrated by studies from our and other groups [16, 32]. Thus, the chronic stress promoted the development of the harmful skeletal muscle changes by enhancing inflammatory actions. A clinical study demonstrated that reductions in the plasma TNF-α and IL-6 values were positively linked to improvements of diagnostic parameters of sarcopenia in HIV-infected individuals who underwent resistance and aerobic exercise training [32]. TNF-α can facilitate cachexic skeletal muscle atrophy and dysfunction in mice bearing a Lewis lung carcinoma tumor [30]. Another animal study reported that TLR-4/MyD88 signaling participates in muscle atrophy [33]. Our present results demonstrated that genetic and pharmacological intervention targeted toward CTSS ameliorated stressed muscle inflammatory actions and muscle dysfunction. Taken together, our findings indicate the ability of CTSS inhibition to mitigate inflammatory over-action, and they suggest that anti-inflammation may exert salutary effects on injured skeletal muscles by TNF-α/TLR-4-MyD88 signaling inactivation, thereby improving morphological alterations under stress conditions.

Experimental and clinical evidence indicates that oxidative stress regulates cytokine/chemokine secretions and proteolytic activity, leading to muscle apoptosis and fibrosis as well as a decline in muscle regeneration [16, 33]. NADPH oxidase is an important source of reactive oxygen species, and a pharmacological inhibition of NADPH oxidase subunits mitigated the development of muscle disease in animals [16, 34]. A significant finding of our present work is that CTSS deletion ameliorated the elevated expressions of the NADPH oxidase subunit genes and/or proteins (p47^phox^ and gp91^phox^), inflammatory genes (TNF-α, ICAM-1, TLR-4, MyD88, and MCP-1), and proteolytic enzymes (CTSK, CTSL, and cystatin C) in the stressed skeletal muscle tissues. Accumulating evidence has shown that inflammatory cytokines can also stimulate targeted proteolytic enzymes’ expression and activation, contributing to skeletal muscle and cardiovascular wall remodeling and fibrosis [16, 18]. CTSS has been shown to act proteolysis outside of the lysosome, similar to the extracellular space [17]. Thus, CTSS increased by oxidative stress and inflammatory cytokines appears to promote stress-induced skeletal muscle injury and remodeling via its non-lysosomal proteolytic function in mice.

The skeletal muscle system contains very rich mitochondria. It has been demonstrated that a decreased expression and acetylation of PGC-1α played a causal role in the mitochondrial and skeletal muscle dysfunction and insulin resistance observed in obese mice with adiponectin receptor deficiency [35]. Accumulating evidence indicates that PGC-1α expression and activity are reduced in diseased muscles of animals and humans [35, 36]. In the present study, stress decreased the PPAR-γ and PGC-1α protein expression, and these changes were rectified by CTSS inhibition. Our findings revealed that CTSS inhibition also mitigated stress-associated mitochondrial injury and lipid droplet accumulation. In vitro, we have shown that CTSS silencing and overexpression, respectively, reduced and increased the stress serum-induced intracellular ROS production and mitochondrial respiration change. Previous research demonstrated that PGC-1α reactivation by increased adiponectin acts as a key mediator of musculoprotective effects of exercise in a senescence-associated mouse prone (SAMP) 10 model [36]. In the same mouse model, we demonstrated that cell therapies with human umbilical cord- and bone marrow-derived mesenchymal stromal cells ameliorate aging-associated skeletal muscle atrophy and dysfunction by modulating PGC-1α-mediated mitochondrial biogenesis [23, 37]. Taken together, the past and present findings indicate that an upregulation of a PPAR-γ axis by CTSS inhibition could contribute to protection against stress-related mitochondrial damage/dysfunction and skeletal muscle dysfunction in mice.

A recent comprehensive review highlighted the roles of members of the cathepsin family in the proliferation and apoptosis of various types of cells [38]. It is notable that CTSS seems to be of particular importance for vascular smooth muscle apoptosis in the vascular repair process. Our observations here show that the lesions of stressed CTSS^–/–^ mice and CTSS inhibitor-treated CTSS^+/+^ mice had lower percentages of TUNEL^+^ apoptotic cells compared to control mice. Because stress induces the activation of CTSS, we favor the hypothesis that CPS promotes muscle loss through its ability to activate elevated CTSS-related skeletal muscle apoptotic activity. It has been known that a Bcl-2/caspase-3 imbalance acts an initiator of cell apoptosis induced by oxidative stress and inflammation [39]. In the present study we observed that the stress-related decreased level of anti-apoptotic Bcl-2 protein and increased levels of pro-apoptotic cleaved caspase-3 protein as well as gp91^phox^ and p47^phox^ genes in the muscles were rectified by the negative modifications of CTSS activity. The stressed muscles had elevated levels of TLR-4 and MyD88 gene; this change was also rectified by CTSS inhibition. The TLR-4/MyD88 axis has been shown to modulate cell apoptosis in vivo and in vitro [40]. Thus, genetic and pharmacological interventions targeted toward CTSS might rectify the alterations in the NAPDH oxidase-mediated oxidative stress and TLR-4/MyD88-mediated inflammatory signaling activation resulting in a Bcl-2/C-caspase-3 imbalance, contributing to the mitigation of muscle apoptosis and muscle mass loss in mice under our experimental conditions. This in vivo concept was further supported by the cellular experimental findings that the silencing and overexpression of CTSS respectively positively and negatively regulate C_2_C_12_ cell apoptosis accompanied by the amelioration of the stress-induced Bcl-2 and C-caspase-3 alterations. It should be noted that CTSS silencing and overexpression, respectively, reduced and increased the stressed serum and oxidative stress-induced the ratio of LC3B-II to LC3B-I, suggesting that CTSS-mediated overactivation of the autophagy-lysosomal pathway might also contribute to the muscle atrophy and dysfunction in mice under our experimental conditions.

There are several study limitations to address. First, the chronic variable stress model used herein is an animal stress model that cannot completely mimic human psychological stress. The study was also not mainly designed to clarify the roles of CTSS in the regulation of inflammation and oxidative stress production in vivo. Second, it remains unclear whether the negative regulation in protein anabolic (IRS-2/Akt-mTOR) and catabolic (MuRF-1/MAFbx1) signaling pathways directly depend on CTSS activity, and we were also unable to identify a mediator to link CTSS activity and protein metabolism imbalance in vivo and in vitro. Moreover, we did not perform a quantitative proteomics analysis (such as tandem mass tag or label-free quantitation) to identify additional proteins or/and microRNA/non-cording RNA that are regulated by CTSS and provide insights into the pathways involved in muscle cell apoptosis and protein turnover. Third, we could not fully explore the role of CTSS in stress-related mitochondrial damage and dysfunction in our experimental mouse model. Forth, we did not investigate not only the short-term effects of CTSS inhibitor treatment on other organs affected by chronic stress, such as the brain and cardiovascular system but also the long-term effects of CTSS inhibitor treatment on muscle function and structure, as well as on overall health and lifespan. Lastly, unfortunately, we have no CTSS overexpressed genetic mice to re-conform our findings. Further research is necessary to investigate these issues.

In conclusion, our findings have demonstrated that the expressions of the CTSS gene and protein were increased in the skeletal muscles of mice subjected to chronic variable stress. CTSS deletion alleviated the skeletal muscle mass loss and remodeling via mitigations of muscle inflammation, oxidative stress production, apoptosis, mitochondrial damage, and the protein anabolic and catabolic imbalance in mice under our experimental stress conditions. The pharmacological inhibition of CTSS mimicked the musculoprotective effect of genetic CTSS deficiency. Recent comprehensive review literature has documented that CTSS activity in health and disease as a treasure trove of untapped clinical potential [41]. Thus, to the best of our knowledge, the present study is the first to report the beneficial effects of the inhibition of CTSS in skeletal muscle atrophy and dysfunction, providing a potential pharmacological therapeutic alternative in the management of muscle diseases in animals under our experimental stress conditions.

### Supplementary Information

Below is the link to the electronic supplementary material.

Supplementary file1 (PDF 1015 KB)

## Data Availability

Not applicable.
