# Supplementary material for: Cathepsin S activity controls chronic stress-induced muscle atrophy and dysfunction in mice
Source: Cell Mol Life Sci. 2023 Aug 17;80(9):254. doi: 10.1007/s00018-023-04888-4 (PMC10435624; doi:10.1007/s00018-023-04888-4)
Supplement: Supplementary file 1 — Supplementary file1 (PDF 1015 KB) [file 18_2023_4888_MOESM1_ESM.pdf]

Supplemental Data

Cathepsin S Activity Controls Chronic Stress-Induced Muscle Atrophy and Dysfunction in Mice

***Corresponding author:** Prof. Xian Wu Cheng, Department of Cardiology and Hypertension, Jilin Provincial Key Laboratory of Stress and Cardiovascular Disease, Yanbian University Hospital, 1327 Juzijie, Yanji 133000, China. Email: chengxw0908@163.com; xianwu@med.nagoya-u.ac.jp

Supplementary Figure Legends

Suppl. Fig. S1. Chronic stress worsened the laminin expression and the desmin expression in gastrocnemius (GAS) muscle of control and stressed $CTSS^{+/+}$ mice at 14 days. **a**,**c:** Representative immunofluorescence images and quantitative data for the intensity of desmin protein expression in GAS muscles of the two experimental groups at Day 14 after stress (n=5). Scale bar: 75 µm. **b**,**d:** A representative TUNEL staining image used to assess the content of apoptotic cells and quantitative data for TUNEL-positive cells (n=6). *Yellow arrows:* $TUNEL^{+}$ cells. **e:** Stress produced a harmful change in targeted mitochondrial biogenesis, oxidative stress-, and inflammation-related gene expression in the GAS muscle of CTSS wildtype ($CTSS^{+/+}$) mice at day 0, 7, and 14 after stress. The data are mean ± SEM, and p-values were determined by one-way ANOVA followed by Tukey's post hoc tests (e) or unpaired Student's t-test (c,d). Control: non-stressed $CTSS^{+/+}$ mice, Stress: 14-day-stressed $CTSS^{+/+}$ mice. *$p<0.05$; **$p<0.01$; N.S, not significant

Suppl. Fig. S2. CTSS deficiency ($CTSS^{-/-}$) mitigated the investigated gene expressions in the GAS muscles of four experimental groups at day 0, 7, and 14 after stress. **a**-**d:** Quantitative polymerase chain reaction (qPCR) data showing the levels of CTSK, CTSL, cystatin C, GLUT-4, PGC-1α, PPAR-γ, $gp91^{phox}$, $p47^{phox}$, TNF-α, ICAM-1, MCP-1, TLR-4 and MyD88 mRNAs. The data are mean ± SEM, and p-values were determined by one-way ANOVA followed by Tukey's post hoc tests. *$p<0.05$; **$p<0.01$; N.S, not significant. CW: $CTSS^{+/+}$ control mice, CK: $CTSS^{-/-}$ control mice, SW: 14-day-stressed $CTSS^{+/+}$ mice, SK: 14-day-stressed $CTSS^{-/-}$ mice.

Suppl. Fig. S3. The cathepsin S inhibitor (CTSS-I) alleviated stress-related muscle fiber microstructure repair and apoptosis in mice. **a**,**b:** Representative immunofluorescence images and quantitative data for the intensity of desmin protein expression in VS and IS groups (n=5 each) at Day 14 after stress. Scale bar: 75 µm. **c:** Representative TUNEL staining used to assess the content of apoptotic cells. **d:** Quantitative data for TUNEL-positive cells in VS and IS mice (n=6). *Yellow arrows:* $TUNEL^{+}$ cells. **e:** Representative immunoblotting images and quantitative data for Desmin and GAPDH protein in the lysates of the GAS muscles of four experimental groups at day 14 after stress (n=3). **f:** CTSS inhibition exerted a beneficial effect on the levels of CTSK, CTSL, cystatin C, GLUT-4, PGC-1α, PPAR-γ, $gp91^{phox}$, $p47^{phox}$, ICAM-1, TLR-4 and MyD88 genes of the GAS muscles of two stressed experimental groups at day 14 after stress. The data are mean ± SEM, and p-values were determined by unpaired Student's t-test (b, d, f) or one-way ANOVA followed by Tukey's post hoc

tests (e). *p<0.05; **p<0.01; N.S, not significant. VC: $CTSS^{+/+}$ mice loaded vehicle; IC: $CTSS^{+/+}$ mice loaded CTSS-I; VS: $CTSS^{+/+}$ mice loaded vehicle+stress; IS: $CTSS^{+/+}$ mice loaded CTSS-I+stress. ig: intragastric administration

Suppl. Fig. S4. CTSS-I ameliorated the investigated molecule alterations in stressed skeletal muscles of four experimental group mice. **a-d:** Representative immunoblotting images and quantitative data for IRS-2, p-PI3K, p-Akt, p-mTOR, p-FoxO1α, MuRF-1, MAFbx1, PGC-1α, PPAR-γ, C-caspase-3, Bcl-2 in GAS muscles at Day 14 after stress (n=3). The data are mean ± SEM, and p-values were determined by one-way ANOVA followed by Tukey's post hoc tests (b-d). VC: $CTSS^{+/+}$ loaded vehicle+non-stress, IC: $CTSS^{+/+}$ loaded CTSS-I+non-stress, VS: $CTSS^{+/+}$ loaded vehicle+stress, IS: $CTSS^{+/+}$ loaded CTSS-I+stress. *p<0.05; **p<0.01; N.S, not significant

Suppl. Fig. S5. Effect of H_2O_2 on C_2C_{12} cell apoptosis. For the evaluation of oxidative stress-induced cell apoptosis, the C_2C_{12} cells were cultured with 0 and 400 μM H_2O_2 for 24 hr, and then were subjected to TUNEL staining. **a**,**b:** Representative image of TUNEL immunofluorescence and combined quantitative data show the numbers of $TUNEL^+$ apoptotic cells treated with 0 and 400 μM H_2O_2 (n=5 each). *Yellow arrows:* $TUNEL^+$ cells. **c-d:** Representative immunoblotting images and quantitative data for CTSS, IRS-2, p-Akt, p-FoxO1α, MuRF-1, C-caspase-3, and Bcl-2 in the lysates of three groups of C_2C_{12} cells (n=3 each). **e:** qPCR analysis of CTSS, CTSK, CTSL and cystatin C gene expressions in C_2C_{12} cells in response to H_2O_2 (n=7, each group). The data are mean ± SEM, and p-values were determined by unpaired Student's t-test (b,d-e) . *p<0.05; **p<0.01; N.S, not significant

Suppl. Fig. S6. CTSS silencing mitigated H_2O_2-induced C_2C_{12} cell apoptosis. C_2C_{12} cells were treated with the non-targeting control RNA and siCTSS for 24 hr and then subjected to the western blotting. **a**: Representative immunoblotting images and quantitative data for the CTSS protein levels of the lysates (n=3 each). Following transfection with non-targeting control RNA and siCTSS for 48 hr, the cells were cultured in serum-free medium containing H_2O_2 at the indicated concentrations for 24 hr. The cells were then subjected to qPCR, apoptosis, and western blotting assays. **b:** qPCR data showing the CTSS expression in the three groups. **c:** *Left panel:* The myotubes with siCTSS or non-targeting control and dose cells treated with H_2O_2 0 μM or H_2O_2 400 μM were subjected to immunofluorescence staining for MHC. *Right panel:* Quantification of the mean myotube diameters. **d:** Representative images of TUNEL immunofluorescence and combined quantitative data showing the numbers of $TUNEL^+$

apoptotic cells in four experimental groups: Cont-H_2O_2-0 μM, si-H_2O_2-0 μM, Cont-H_2O_2-400 μM, and si-H_2O_2-400 μM (n=5 each). *Yellow arrows:* $TUNEL^+$ cells. Scale bar: 200 μm. **e-f:** Representative immunoblotting images and quantitative data for CTSS, IRS-2, p-Akt, p-FoxO1α, MuRF-1, C-caspase-3, and Bcl-2 in whole-cell lysates of four groups of C_2C_{12} cells (n=3). The data are mean ± SEM, and p-values were determined by one-way ANOVA followed by Tukey's post hoc tests (b,c,f) or unpaired Student's t-test (a,d). *p<0.05; **p<0.01; N.S, not significant

Suppl. Fig. S7. CTSS overexpression enhanced H_2O_2-induced C_2C_{12} cell apoptosis. C_2C_{12} cells were treated with control empty plasma and pl-CTSS for 24 hr and then subjected to a western blotting assay. **a:** Representative immunoblotting images and quantitative data for the CTSS protein levels of two experimental groups (n=3 each). Following transfection with control empty plasma and pl-CTSS for 48 hr, the cells were cultured in serum-free medium containing H_2O_2 at the indicated concentrations for 24 hr and then subjected to qPCR, apoptosis, and western blotting assays. **b:** qPCR data showing the CTSS expression in three groups. **c:** Representative images show green fluorescent protein (GFP)-labeled CTSS $plasmid^+$ C_2C_{12} cells after transfection, at 0 hr and 16 hr. Scale bar: 500 μm. **d:** *Upper panel:* The myotubes with pl-CTSS or control empty plasma and dose cells were treated with H_2O_2 0 μM or H_2O_2 400 μM and then subjected to immunofluorescence staining for MHC. *Lower panel:* Quantification of the mean myotube diameters of four experimental groups: Cont-H_2O_2-0 μM, pl-H_2O_2-0 μM, Cont-H_2O_2-400 μM, and pl-H_2O_2-400 μM (n=5 each). **e:** Representative images of TUNEL immunofluorescence and combined quantitative data showing the numbers of $TUNEL^+$ apoptotic cells in the four experimental groups: Cont-H_2O_2-0 μM, pl-H_2O_2-0 μM, Cont-H_2O_2-400 μM, and pl-H_2O_2-400 μM (n=5 each). *Yellow arrows:* $TUNEL^+$ cells Scale bar: 200 μm. **f:** Representative immunoblotting images and quantitative data for CTSS, IRS-2, p-Akt, p-FoxO1α, MuRF-1, C-caspase-3, and Bcl-2 in whole-cell lysates of four groups of C_2C_{12} cells (n=3 each). The data are mean ± SEM, and p-values were determined by one-way ANOVA followed by Tukey's post hoc tests (b,d,f) or unpaired Student's t-test (a,e). *p<0.05; **p<0.01; N.S, not significant

Suppl. Fig. S8 The mechanism underlying CTSS-mediated IRS-2 signaling inactivation in stress-induced skeletal muscle wasting and dysfunction. Abbreviation: Bcl-2, apoptosis-related B-cell lymphoma 2; IRS-2, insulin receptor substrate 2; MAFbx1, muscle RING-finger protein-1 protein; MuRF-1, forkhead box-1; p-Akt, phospho-protein kinase B; p-mTOR, phospho-mammalian target of rapamycin; p-PI3K, phospho-phosphatidylinositol 3-kinase; PPAR-α; peroxisome proliferator-activated

receptor-γ coactivator-α.

Table S1. The information of experiment groups

<table>
<tr><td colspan="5">a. For time course</td></tr>
<tr><td colspan="2">Group name</td><td>CTSS types</td><td>Stress days</td><td>n=</td></tr>
<tr><td colspan="2">Non-stress</td><td>CTSS$^{+/+}$ mice</td><td>0</td><td>7</td></tr>
<tr><td colspan="2">Stress-7days</td><td>CTSS$^{+/+}$ mice</td><td>7</td><td>7</td></tr>
<tr><td colspan="2">Stress-14days</td><td>CTSS$^{+/+}$ mice</td><td>14</td><td>7</td></tr>
<tr><td colspan="5">b. For CTSS deficiency function detection</td></tr>
<tr><td colspan="2">Group name</td><td>Mice types</td><td>Stress days</td><td>n=</td></tr>
<tr><td colspan="2">CW</td><td>CTSS$^{+/+}$ mice</td><td>0</td><td>8</td></tr>
<tr><td colspan="2">CK</td><td>CTSS$^{-/-}$ mice</td><td>0</td><td>8</td></tr>
<tr><td colspan="2">SW</td><td>CTSS$^{+/+}$ mice</td><td>14</td><td>8</td></tr>
<tr><td colspan="2">SK</td><td>CTSS$^{-/-}$ mice</td><td>14</td><td>8</td></tr>
<tr><td colspan="5">c. For Pharmacological CTSS inhibition function (CTSS$^{+/+}$ mice)</td></tr>
<tr><td>Group name</td><td>CTSS</td><td>Stress days</td><td>Intragastric administration</td><td>n=</td></tr>
<tr><td>VC</td><td>CTSS$^{+/+}$ mice</td><td>0</td><td>vehicle</td><td>8</td></tr>
<tr><td>IC</td><td>CTSS$^{+/+}$ mice</td><td>0</td><td>CTSS inhibitor</td><td>8</td></tr>
<tr><td>VS</td><td>CTSS$^{+/+}$ mice</td><td>14</td><td>vehicle</td><td>8</td></tr>
<tr><td>IS</td><td>CTSS$^{+/+}$ mice</td><td>14</td><td>CTSS inhibitor</td><td>8</td></tr>
</table>

Table S2: Primer sequences for mice used for quantitative real-time PCR

Genes	Forward Primers	Reverse Primers
CTSS	GTGGCCACTA AAGGGCCTG	ACCGCTTTTGTAGAAGAAGAAGGAG
CTSK	AGCAGGCTGGAGGACTAAGGT	TTTGTGCATCTCAGTGGAAGACT
CTSL	ACAGAAGACTGTATGGCACGA	GTATTCCCCGTTGTGTAGCTG
cystatin C	TGAGCGAGTACAACAAGGGC	GGCTGGTCATGGAAAGGACAG
gp91^{phox}	ACTTTCCATAAGATGGTAGCTTGG	GCATTCACACACCACTCAACG
p47^{phox}	AACTACCTGGAGCCAGTTGAG	AATTAGGAGGTGGTGGAATATCGG
GLUT-4	ACACTGGTCCTAGCTGTATTCT	CCAGCCACGTTGCATTGTA
PGC1α	AGACGGATTGCCCTCATTTGA	GGTCTTAACAATGGCAGGGTTT
PPAR-γ	GGAAGACCACTCGCATTCCTT	GTAATCAGCAACCATTGGGTCA
TNF-α	CAGGCGGTGCCTATGTCTC	CGATCACCCCGAAGTTCAGTAG
ICAM-1	CCCCGCAGGTCCAATTC	CCAGAGCGGCAGAGCAA
MCP-1	GCCCCACTCACCTGCTGCTACT	CCTGCTGCTGGTGATCCTCTTGT
TLR-4	AGTGGGTCAAGGAACAGAAGCA	CTTTACCAGCTCATTTCTCACC
MyD88	TCATGTTCTCCATACCCTTGGT	AAACTGCGAGTGGGGTCAG
GAPDH	ATGTGTCCGTCGTGGATCTGA	ATGCCTGCTTCACCACCTTCT

Abbreviations: CTSS (cathepsin S); CTSK (cathepsin K); CTSL (cathepsin L); cystatin C; gp91^{phox}; p47^{phox}; GLUT-4 (Glucose Transporter 4); PGC1α (peroxisome proliferator-activated receptor co-activator-1α); PPAR-γ (peroxisome proliferator-activated receptor-γ); TNF-α (tumor necrosis factor-α); ICAM-1 (Vascular cell adhesion molecule-1); MCP-1 (monocyte chemoattractant protein-1); TLR-4 (toll-like receptor-4); MyD88 (myeloid differentiation primary response 88), GAPDH (Glyceraldehyde-3-phosphate dehydrogenase

Fig S1

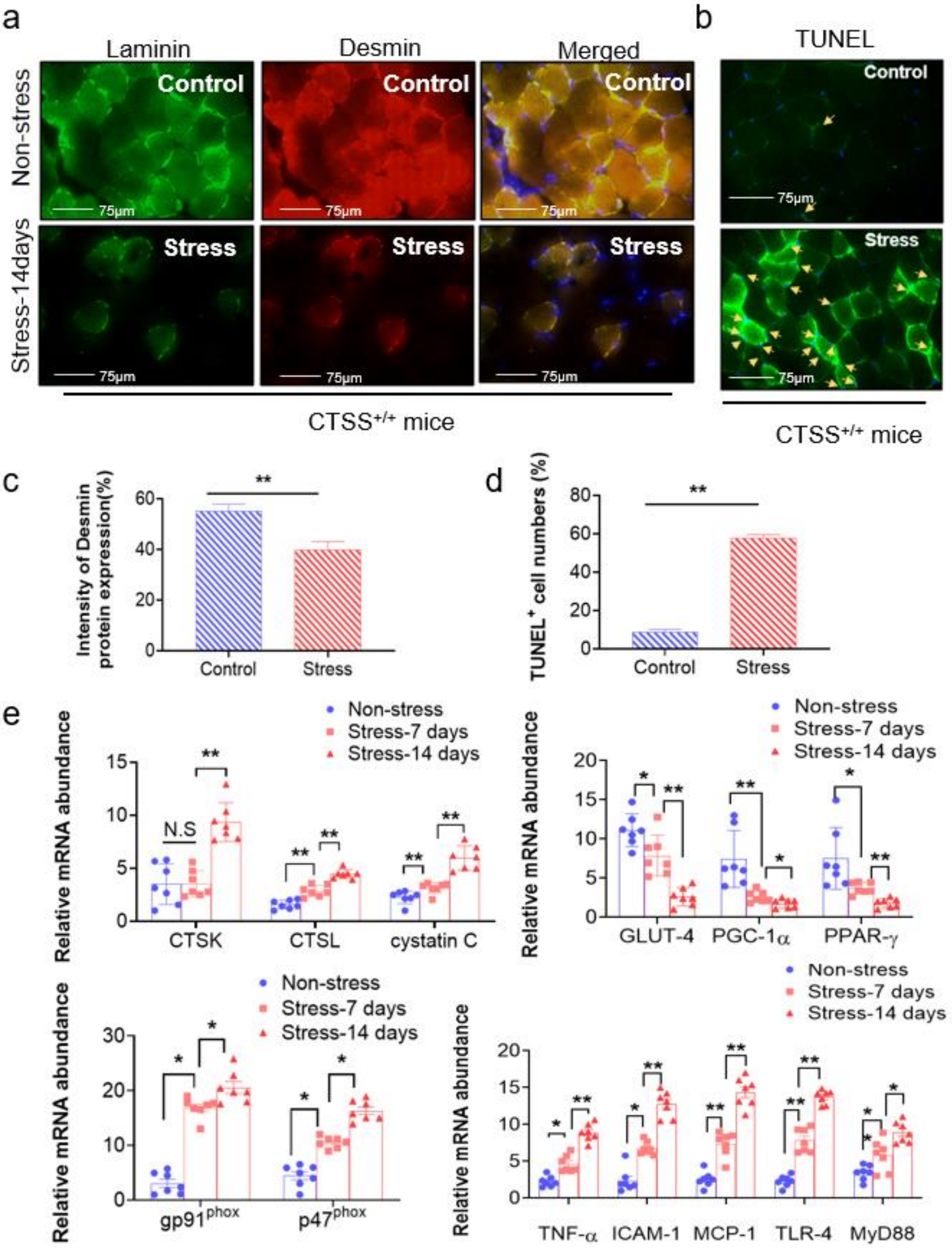
a
Laminin
Desmin
Merged
Non-stress
Stress-14days
Control
Stress
75μm
CTSS+/+ mice
b
TUNEL
Control
Stress
75μm
CTSS+/+ mice
c
Intensity of Desmin protein expression(%)
Control
Stress
d
TUNEL+ cell numbers (%)
Control
Stress
e
Non-stress
Stress-7 days
Stress-14 days
Relative mRNA abundance
N.S
CTSK
CTSL
cystatin C
GLUT-4
PGC-1α
PPAR-γ
gp91phox
p47phox
TNF-α
ICAM-1
MCP-1
TLR-4
MyD88

Fig S2

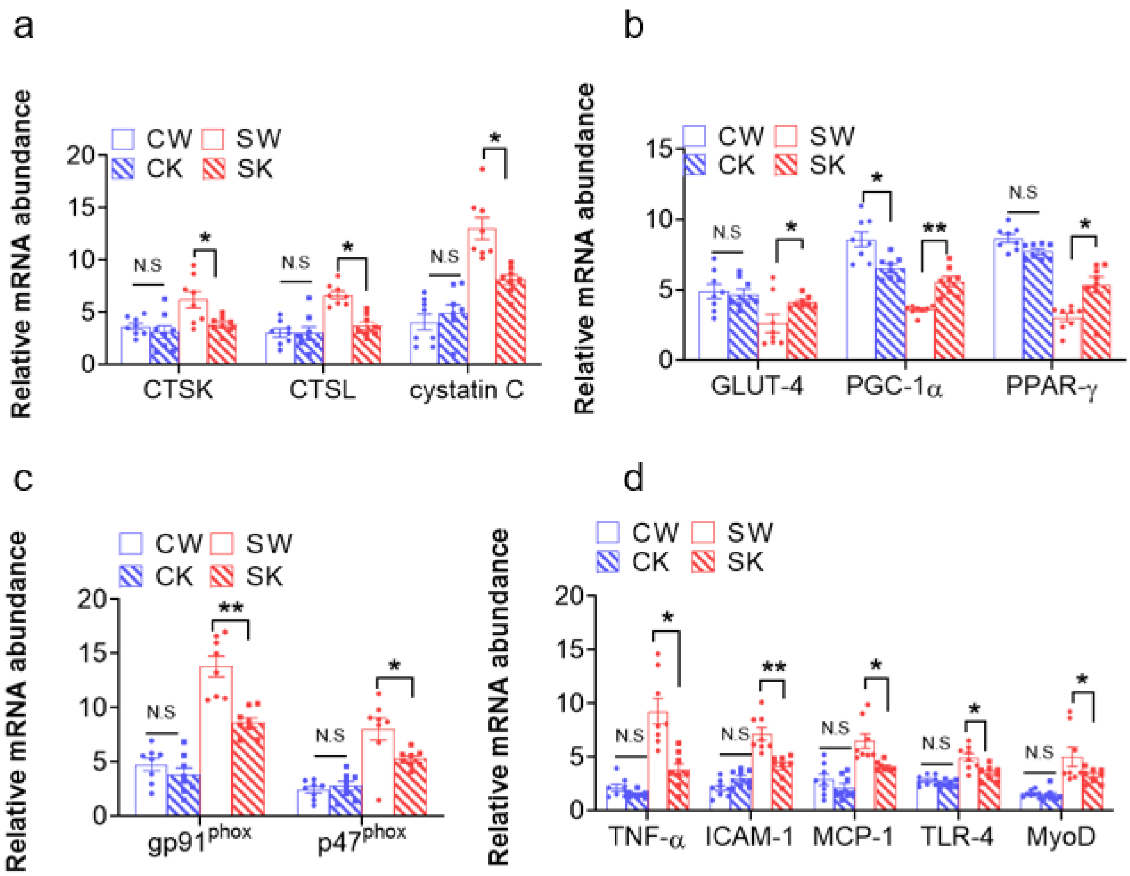
a
b
c
d
Relative mRNA abundance
CW
SW
CK
SK
N.S
CTSK
CTSL
cystatin C
GLUT-4
PGC-1α
PPAR-γ
gp91phox
p47phox
TNF-α
ICAM-1
MCP-1
TLR-4
MyoD

Fig S3

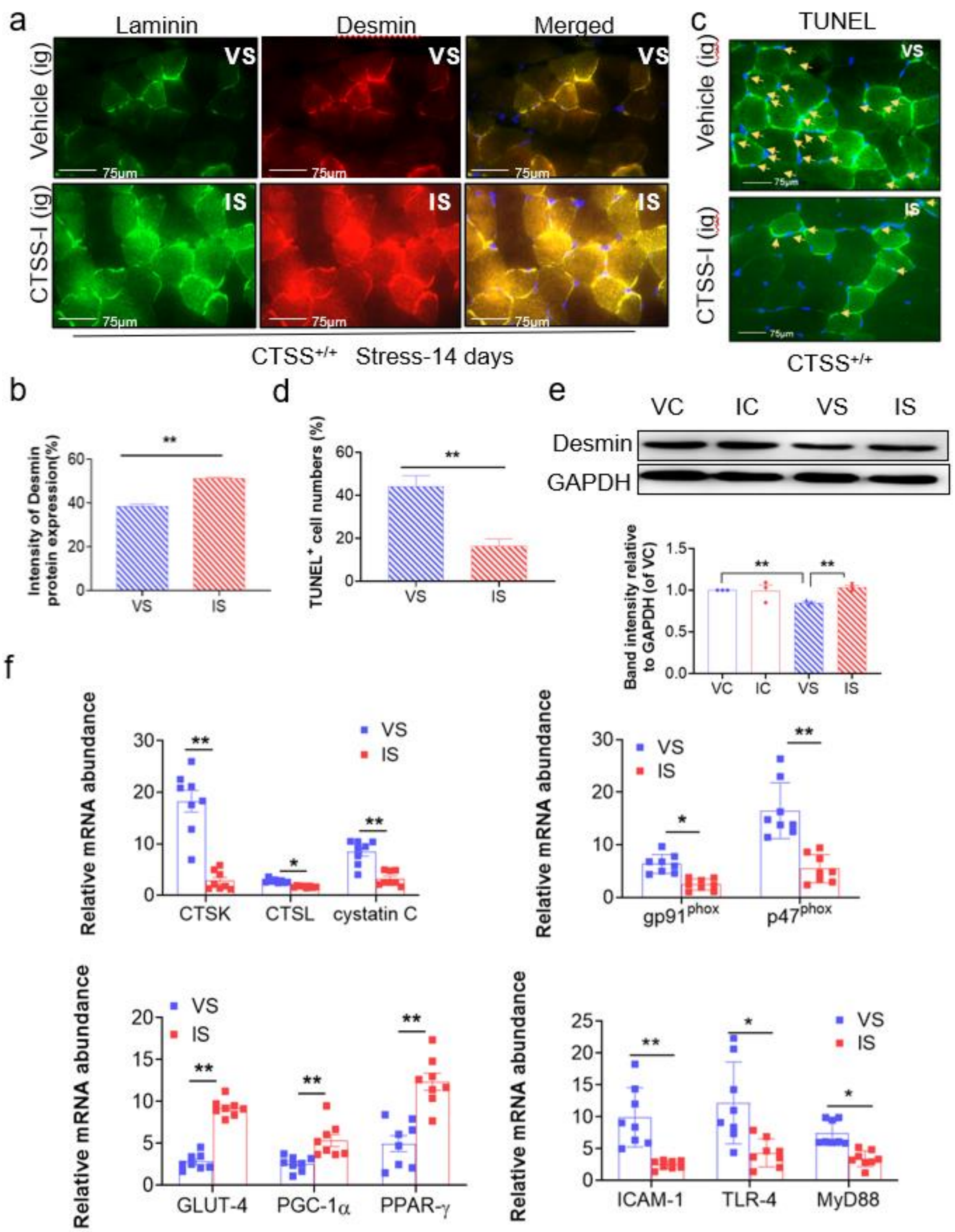
a
Laminin
Desmin
Merged
Vehicle (ig)
CTSS-I (ig)
VS
IS
75μm
CTSS+/+ Stress-14 days
c
TUNEL
CTSS+/+
b
Intensity of Desmin protein expression(%)
d
TUNEL+ cell numbers (%)
e
VC
IC
VS
IS
Desmin
GAPDH
Band intensity relative to GAPDH (of VC)
f
Relative mRNA abundance
CTSK
CTSL
cystatin C
gp91phox
p47phox
GLUT-4
PGC-1α
PPAR-γ
ICAM-1
TLR-4
MyD88

Fig S4

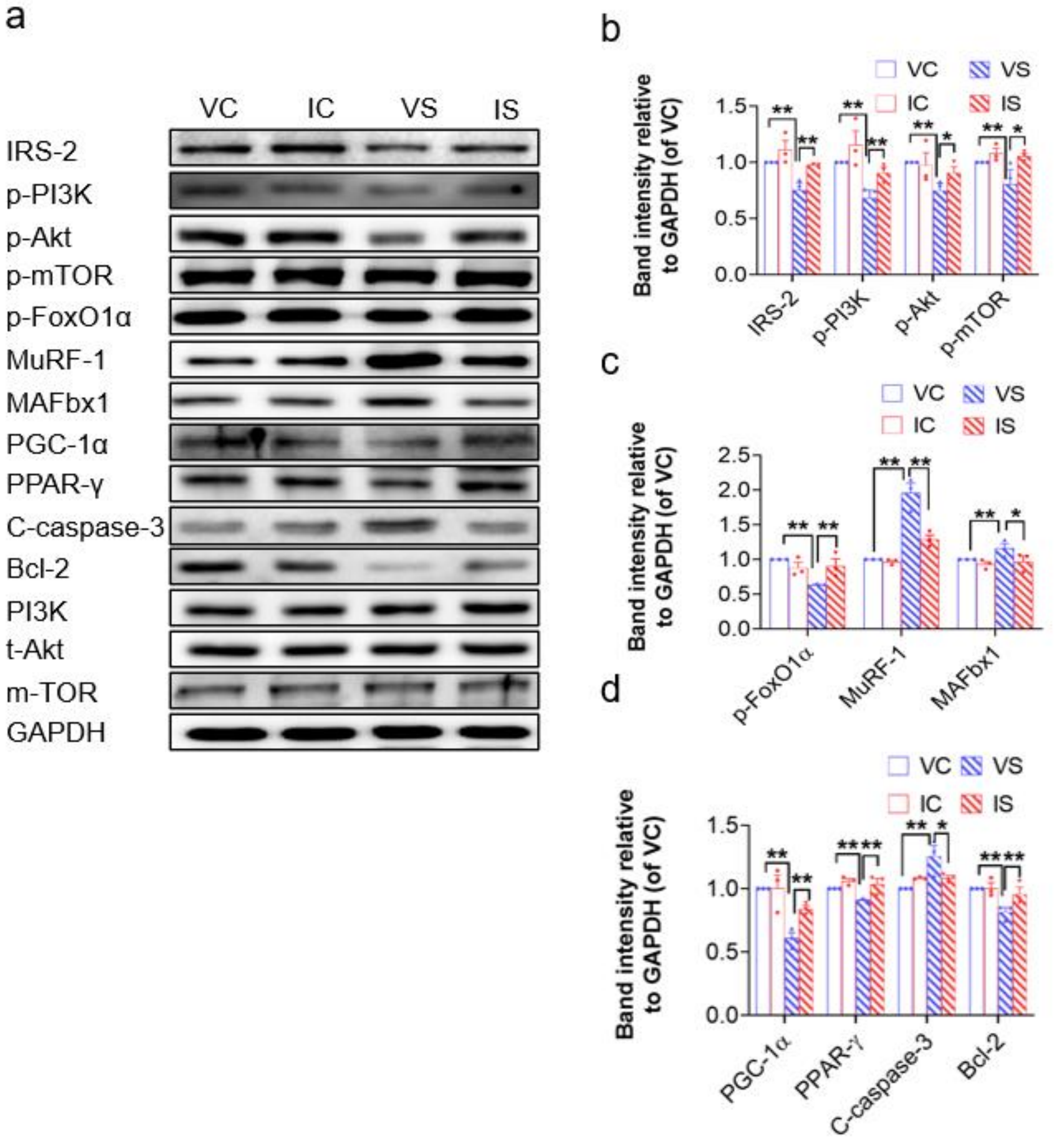
a
VC
IC
VS
IS
IRS-2
p-PI3K
p-Akt
p-mTOR
p-FoxO1α
MuRF-1
MAFbx1
PGC-1α
PPAR-γ
C-caspase-3
Bcl-2
PI3K
t-Akt
m-TOR
GAPDH
b
Band intensity relative to GAPDH (of VC)
VC
VS
IC
IS
IRS-2
p-PI3K
p-Akt
p-mTOR
c
p-FoxO1α
MuRF-1
MAFbx1
d
PGC-1α
PPAR-γ
C-caspase-3
Bcl-2

Fig S5

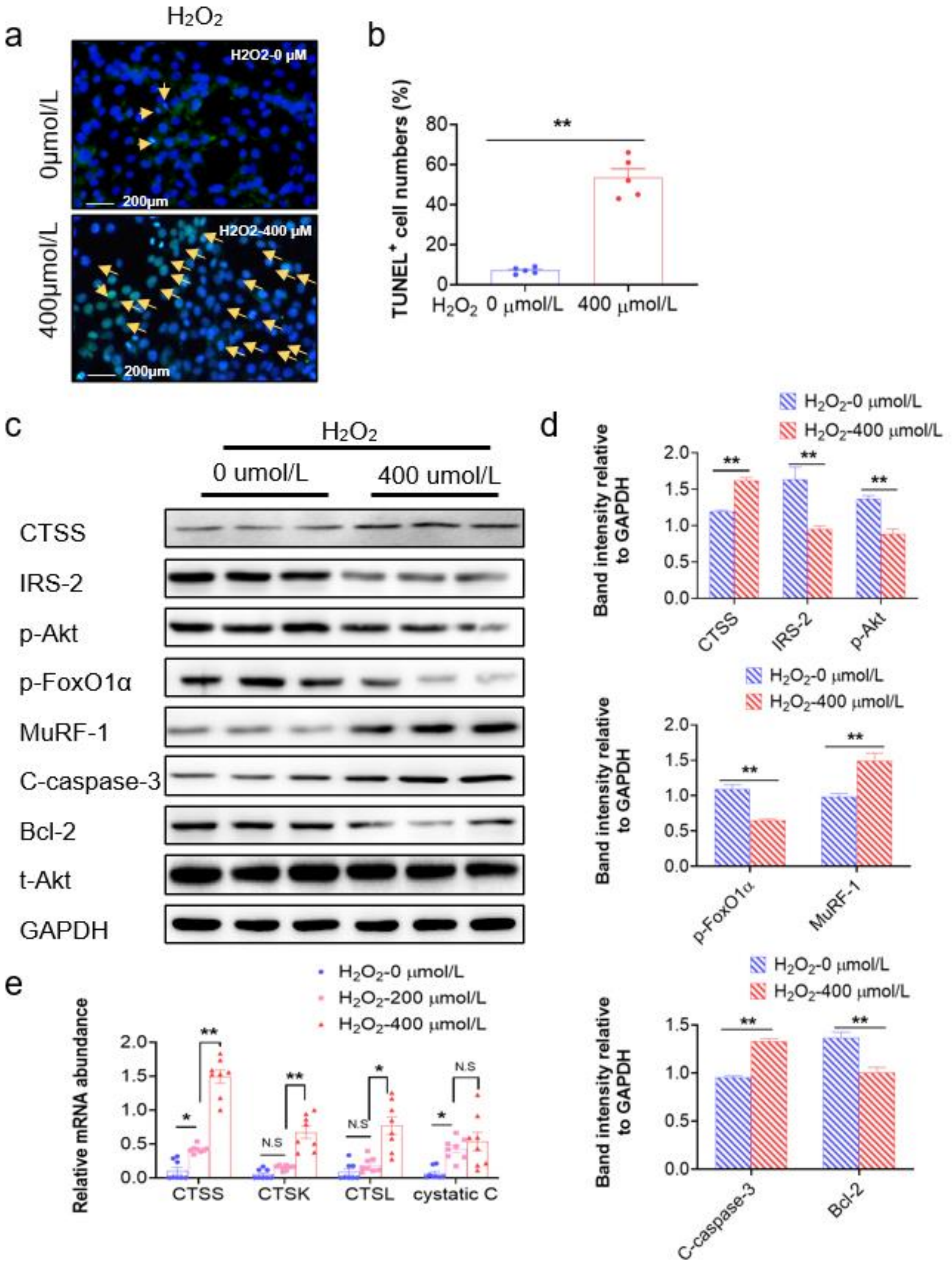

a
H2O2
0μmol/L
400μmol/L
H2O2-0 μM
H2O2-400 μM
200μm
b
TUNEL+ cell numbers (%)
**
H2O2 0 μmol/L 400 μmol/L
c
H2O2
0 umol/L
400 umol/L
CTSS
IRS-2
p-Akt
p-FoxO1α
MuRF-1
C-caspase-3
Bcl-2
t-Akt
GAPDH
d
H2O2-0 μmol/L
H2O2-400 μmol/L
Band intensity relative to GAPDH
CTSS
IRS-2
p-Akt
p-FoxO1α
MuRF-1
C-caspase-3
Bcl-2
e
H2O2-0 μmol/L
H2O2-200 μmol/L
H2O2-400 μmol/L
Relative mRNA abundance
CTSS
CTSK
CTSL
cystatic C
N.S

Fig S6

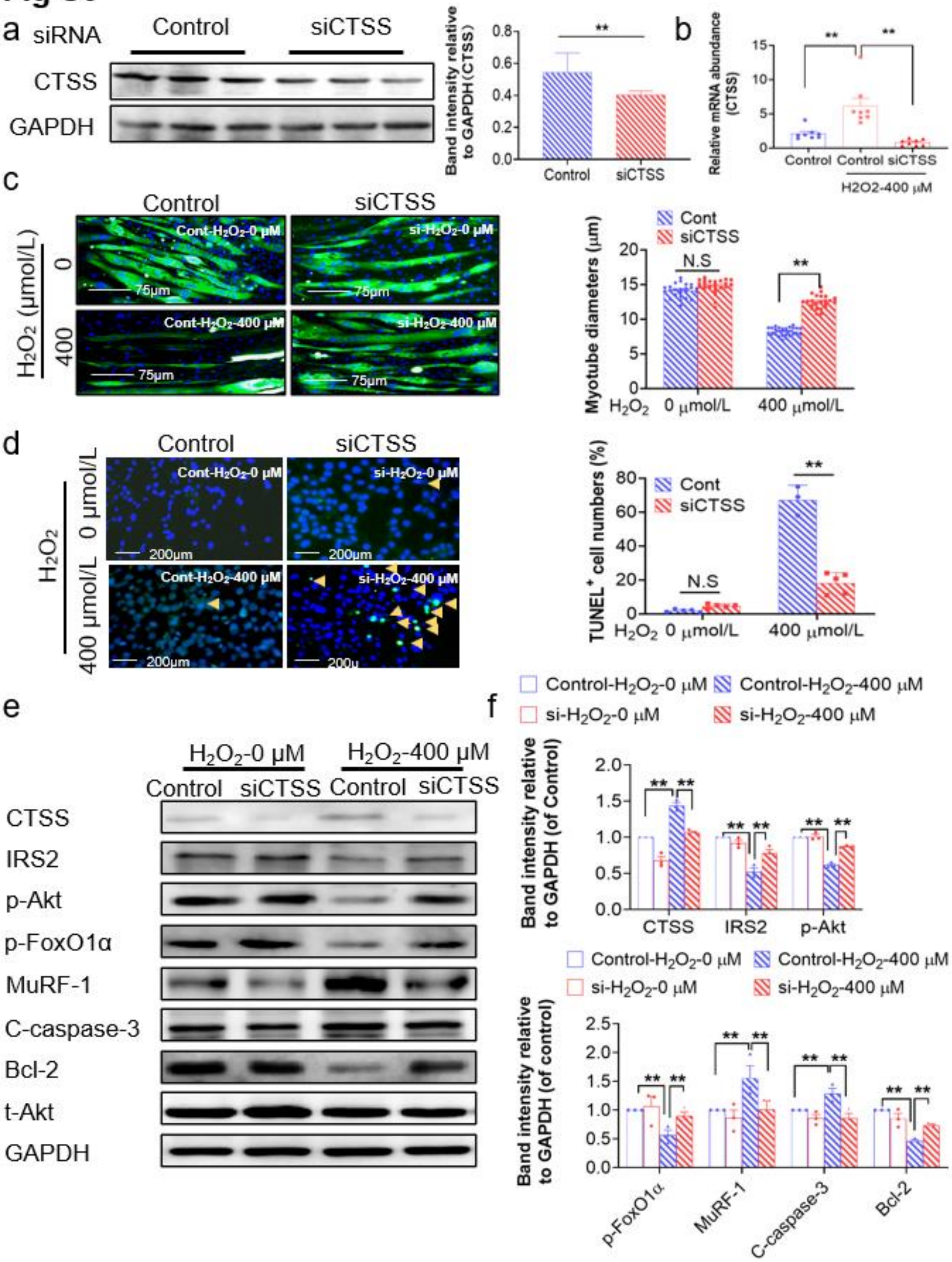

a siRNA
Control
siCTSS
CTSS
GAPDH
Band intensity relative to GAPDH (CTSS)
b
Relative mRNA abundance (CTSS)
H2O2-400 μM
c
H$_2$O$_2$ (μmol/L)
Cont-H$_2$O$_2$-0 μM
si-H$_2$O$_2$-0 μM
Cont-H$_2$O$_2$-400 μM
si-H$_2$O$_2$-400 μM
75μm
Myotube diameters (μm)
Cont
N.S
d
TUNEL$^+$ cell numbers (%)
200μm
0 μmol/L
400 μmol/L
e
H$_2$O$_2$-0 μM
H$_2$O$_2$-400 μM
IRS2
p-Akt
p-FoxO1α
MuRF-1
C-caspase-3
Bcl-2
t-Akt
f
Control-H$_2$O$_2$-0 μM
Control-H$_2$O$_2$-400 μM
si-H$_2$O$_2$-0 μM
si-H$_2$O$_2$-400 μM
Band intensity relative to GAPDH (of Control)

Fig S7

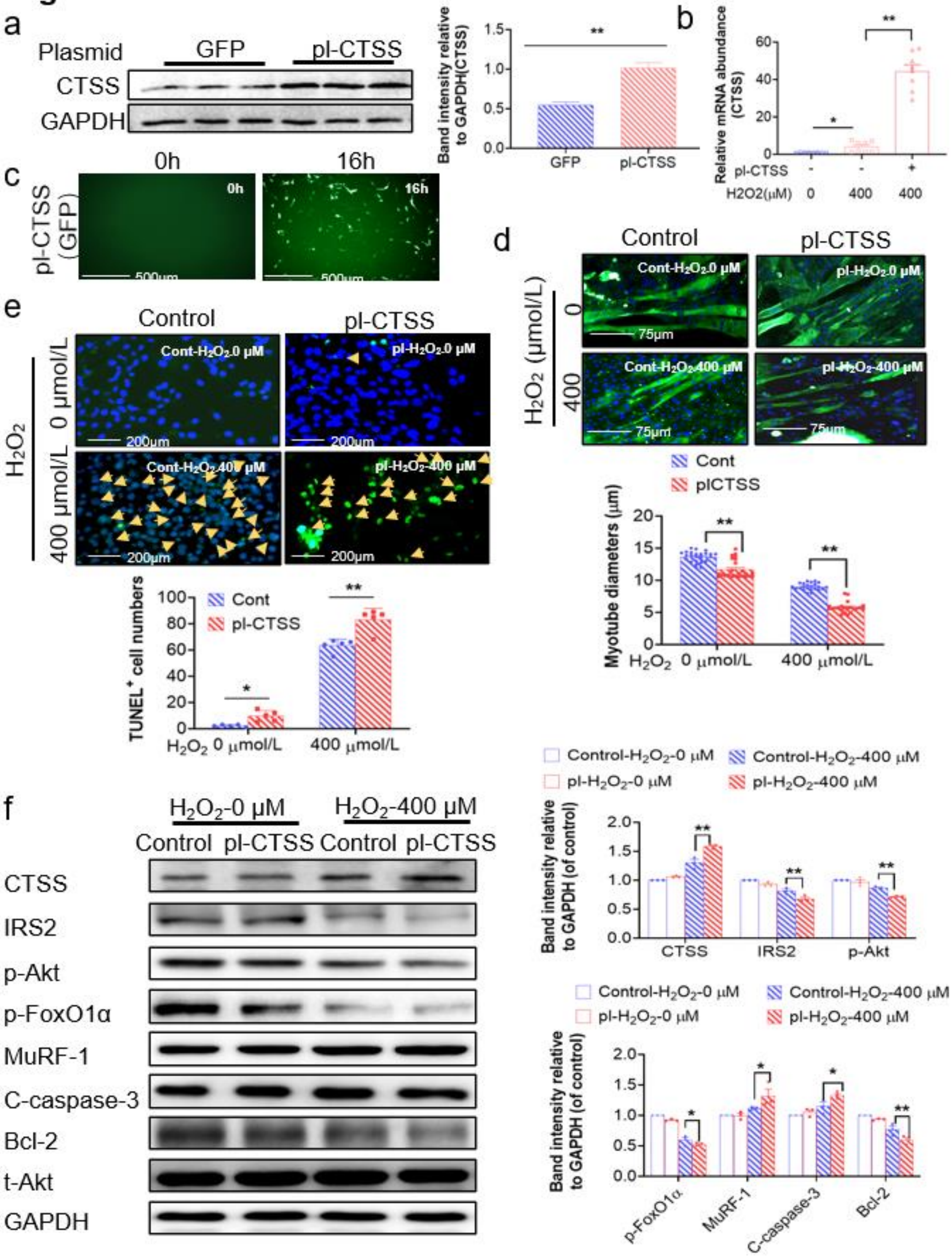

a
Plasmid
GFP
pl-CTSS
CTSS
GAPDH
Band intensity relative to GAPDH(CTSS)
GFP
pl-CTSS
b
Relative mRNA abundance (CTSS)
pl-CTSS
H2O2(μM)
c
0h
16h
pl-CTSS (GFP)
500μm
d
Control
pl-CTSS
H2O2 (μmol/L)
Cont-H2O2 0 μM
pl-H2O2 0 μM
Cont-H2O2 400 μM
pl-H2O2 400 μM
75μm
Cont
plCTSS
Myotube diameters (μm)
H2O2 0 μmol/L
400 μmol/L
e
Control
pl-CTSS
H2O2
0 μmol/L
400 μmol/L
200μm
Cont
pl-CTSS
TUNEL+ cell numbers
f
H2O2-0 μM
H2O2-400 μM
Control
pl-CTSS
CTSS
IRS2
p-Akt
p-FoxO1α
MuRF-1
C-caspase-3
Bcl-2
t-Akt
GAPDH
Control-H2O2-0 μM
Control-H2O2-400 μM
pl-H2O2-0 μM
pl-H2O2-400 μM
Band intensity relative to GAPDH (of control)

Fig S8

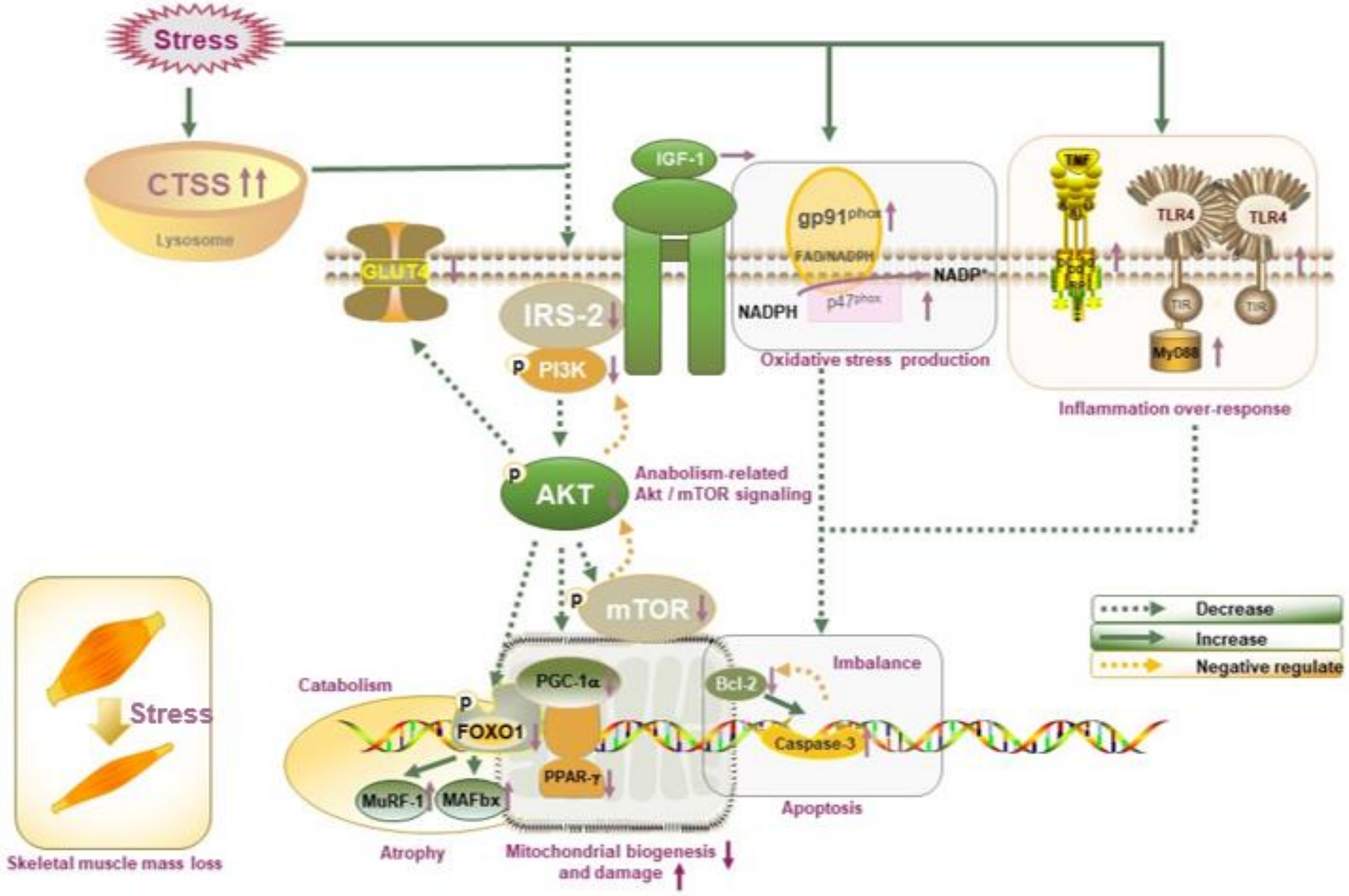
Stress
CTSS
Lysosome
GLUT4
IRS-2
PI3K
IGF-1
gp91phox
FAD/NADPH
NADP+
NADPH
p47phox
Oxidative stress production
TNF
TLR4
TLR4
TIR
TIR
MyD88
Inflammation over-response
AKT
Anabolism-related
Akt / mTOR signaling
mTOR
Stress
Skeletal muscle mass loss
Catabolism
FOXO1
MuRF-1
MAFbx
Atrophy
PGC-1α
PPAR-γ
Mitochondrial biogenesis
and damage
Bcl-2
Imbalance
Caspase-3
Apoptosis
Decrease
Increase
Negative regulate
